# Reduction in Long COVID Symptoms and Symptom Severity in Vaccinated Compared to Unvaccinated Adults

**DOI:** 10.1093/ofid/ofae039

**Published:** 2024-01-23

**Authors:** Hannah E Maier, Theresa Kowalski-Dobson, Ashley Eckard, Carmen Gherasim, David Manthei, Alyssa Meyers, Dawson Davis, Kevin Bakker, Kathleen Lindsey, Zijin Chu, Lauren Warsinske, Matthew Arnold, Anna Buswinka, Emily Stoneman, Riccardo Valdez, Aubree Gordon

**Affiliations:** Department of Epidemiology, School of Public Health, University of Michigan, Ann Arbor, Michigan, USA; Department of Epidemiology, School of Public Health, University of Michigan, Ann Arbor, Michigan, USA; Department of Epidemiology, School of Public Health, University of Michigan, Ann Arbor, Michigan, USA; Department of Pathology, Michigan Medicine, University of Michigan, Ann Arbor, Michigan, USA; Department of Pathology, Michigan Medicine, University of Michigan, Ann Arbor, Michigan, USA; Department of Epidemiology, School of Public Health, University of Michigan, Ann Arbor, Michigan, USA; Department of Pathology, Michigan Medicine, University of Michigan, Ann Arbor, Michigan, USA; Department of Epidemiology, School of Public Health, University of Michigan, Ann Arbor, Michigan, USA; Department of Epidemiology, School of Public Health, University of Michigan, Ann Arbor, Michigan, USA; Department of Epidemiology, School of Public Health, University of Michigan, Ann Arbor, Michigan, USA; Department of Epidemiology, School of Public Health, University of Michigan, Ann Arbor, Michigan, USA; Department of Epidemiology, School of Public Health, University of Michigan, Ann Arbor, Michigan, USA; Department of Epidemiology, School of Public Health, University of Michigan, Ann Arbor, Michigan, USA; Department of Internal Medicine, Michigan Medicine, University of Michigan, Ann Arbor, Michigan, USA; Department of Pathology, Michigan Medicine, University of Michigan, Ann Arbor, Michigan, USA; Department of Epidemiology, School of Public Health, University of Michigan, Ann Arbor, Michigan, USA

**Keywords:** community-based, COVID-19, long COVID, SARS-CoV-2, vaccine

## Abstract

**Background:**

The impact of vaccination prior to infection on postacute sequelae of coronavirus disease 2019 (COVID-19, PASC), also known as long COVID, remains unclear. Here we assess the protective effect of vaccination on long COVID in a community-based setting.

**Methods:**

The Immunity Associated with SARS-CoV-2 (IASO) study is an ongoing prospective cohort of working adults that began in October 2020. Participants are actively followed for severe acute respiratory syndrome coronavirus 2 infection. We compared the prevalence of symptoms and symptom severity in vaccinated compared to unvaccinated cases. Our primary definition of long COVID was the presence of symptoms at 90 days postinfection; 30 days postinfection was also examined.

**Results:**

Overall, by 90 days postinfection, 13% of cases had long COVID, with 27% of unvaccinated cases and 8% of vaccinated cases reporting long COVID (relative risk [RR], 0.31 [95% confidence interval {CI}, .22–.42]). Vaccination was also associated with significantly lower average severity scores at all timepoints (eg, relative severity at 90 days postinfection: −2.70 [95% CI, −1.68 to −3.73]). In the pre-Omicron era, 28% of unvaccinated cases and 18% of vaccinated cases reported long COVID (*P* = .07), and vaccinated cases reported less severe symptoms including less difficulty breathing (*P* = .01; 90-day RR, 0.07).

**Conclusions:**

Vaccinated cases had lower prevalence of long COVID and reduced symptom severity.

## Teaser

In a prospective, community-based cohort study of adults, we identified SARS-CoV-2 infections and examined the presence of postacute sequelae of COVID-19 (long COVID). Vaccination was associated with lower prevalence and severity of long COVID symptoms.

Over the last 3 years the substantial burden of long-term effects for nonfatal coronavirus disease 2019 (COVID-19) cases including long-lasting symptoms and postacute sequelae of COVID-19 (called PASC or long COVID) has become apparent. In the United States (US), the Centers for Disease Control and Prevention’s (CDC) household pulse survey reports that 15% of all US adults have ever experienced long COVID symptoms 3 or more months after infection (∼38 million adults, based on 2020 census), and 6% of US adults (∼15 million adults) are currently experiencing long COVID [1, 2].

Severe acute illness is associated with long COVID, and illness severity has varied by viral strain. [3], with a study showing that the Omicron variant may be associated with less long COVID [4]. The Delta SARS-CoV-2 variant causes more severe acute illness than previous strains [5], while Omicron has been thought to be less severe than prior variants [6, 7]. Generally, much less is known about long COVID following mild or moderate SARS-CoV-2 infections, and thus the full spectrum of disease, because a majority of the studies are hospital-based.

While studies have investigated the effects of vaccination on long COVID, most are hospital-based or use electronic health record data, both of which are subject to various biases. Because of biases, the existing studies on vaccination and long COVID may not present accurate estimates of the prevalence of long COVID or the potential benefits of vaccination for the general adult population [8–17]. Here we use an ongoing community-based cohort study to examine the impact of vaccination on long COVID.

## METHODS

### Patient Consent Statement

Written consent was obtained for all participants. The study was approved by the institutional review board at the University of Michigan (UM) Medical School (HUM00184533).

### Study Design and Enrollment

The Immunity Associated with SARS-CoV-2 (IASO; named for the Greek goddess of recuperation from illness) study is an ongoing prospective cohort study that began enrollment in October 2020 [18].

Initial enrollment criteria were adults aged 18 years or older who were employees at UM or professional students. Participants were initially enrolled for 1 year, then invited to reenroll on a yearly basis. Demographic information and SARS-CoV-2 history were collected at enrollment. Participants complete weekly symptom and respiratory illness testing surveys and have blood samples collected every 2 months. In addition, bimonthly surveys collect information on in-person work and social distancing. All surveys are conducted online through REDCap.

### Case Identification

Cases were defined as those that had a documented or reported positive SARS-CoV-2 respiratory virus test (either molecular or rapid antigen test), regardless of symptoms. At enrollment, participants were encouraged to reach out to study staff and/or schedule a SARS-CoV-2 test through UM at the first sign of illness. Clinical molecular SARS-CoV-2 testing was provided to participants free of charge through the UM Health System. Participants who reported any symptoms in the weekly symptom and respiratory illness testing surveys were asked about SARS-CoV-2 testing already completed, prompted to schedule testing, and were able to indicate if they wanted a study nurse to follow up with them to schedule testing. Participants who did not report any symptoms were also asked about SARS-CoV-2 testing, results, and reason for the test(s) if performed; many participants tested if they were exposed and some participated in a weekly community testing program. Participants were asked to submit documentation for outside testing including photos of home rapid tests. SARS-CoV-2 testing data from UM medical records was collected weekly to identify any testing performed at UM that was not reported to the study team. When a new SARS-CoV-2 illness was identified, participants were offered weekly serial respiratory testing through the UM clinical testing site weekly until negative twice and were asked to contribute a blood sample 3–4 weeks post–illness onset; all consecutive positive tests were considered as belonging to a single COVID-19 episode. SARS-CoV-2 infections are verified by the study team using 2 lines of evidence when possible. Possible sources of evidence include self-reported rapid antigen tests, diagnostic nucleic acid amplification testing, viral sequencing, serial polymerase chain reaction monitoring, and immunoassays for SARS-CoV-2 spike or nucleocapsid antibodies.

### Vaccination Data and Confirmation

Vaccination data, including the vaccine type and date, were systematically recorded. Sources of vaccination data included participant surveys, UM medical records, and the Michigan Care Improvement Registry, the state of Michigan’s vaccine registry. Participants reporting vaccinations that did not match their UM medical record or the vaccine registry were asked to submit documentation of immunization. Vaccination was required for nearly all UM employees and students as of August 2021 and boosters were required as of January 2022.

### Acute Symptoms and Postacute Sequelae of SARS-CoV-2 Infection

PASC illness surveys were sent out at 30 days postinfection to everyone with a symptomatic SARS-CoV-2 acute infection. PASC surveys collected information about the presence, duration, and severity (on a scale of 1 to 10) of a set of 15 symptoms commonly reported with SARS-CoV-2 infections (sore throat, nasal congestion, cough, muscle or body aches, fever/feverishness, fatigue, chills, difficulty breathing, chest pressure, sleep issues, loss of taste, loss of smell, headaches, gastrointestinal [GI] issues, cognitive dysfunction) during the acute infection and at 30 days. If a participant was still symptomatic at the initial survey, PASC surveys were sent every 30 days until symptoms resolved. PASC surveys began in June 2021; prior infections were sent surveys retroactively.

### Exposures

Immunity status prior to infection was defined as naive (no prior infection or vaccination), breakthrough (fully vaccinated ≥14 days prior to infection), unvaccinated with prior infection, and those between vaccine doses. For breakthrough infections, additional exposures were considered: Omicron (infection after 25 December 2021) versus pre-Omicron variants, timing since last vaccination (≥6 months vs <6 months prior), and prior infection versus no prior infection.

### Statistical Analysis

We compared the presence and severity of self-reported acute and long-term SARS-CoV-2 symptoms between groups. Poisson regression models were used to calculate relative risks (prevalence ratios) comparing the likelihood of having symptoms between groups. Linear regression models were used to compare symptom severity between groups. When there were zero counts for a symptom in one group, Fisher exact tests were used to assess significance and 0.5 was added to all 4 cells (levels of exposure [yes/no] and outcome [yes/no]) to calculate the relative risk (RR). Analysis was performed using R software, version 4.3.0 (R Foundation for Statistical Computing).

## RESULTS

### Study Participation and SARS-CoV-2 Cases

**Figure 1.**
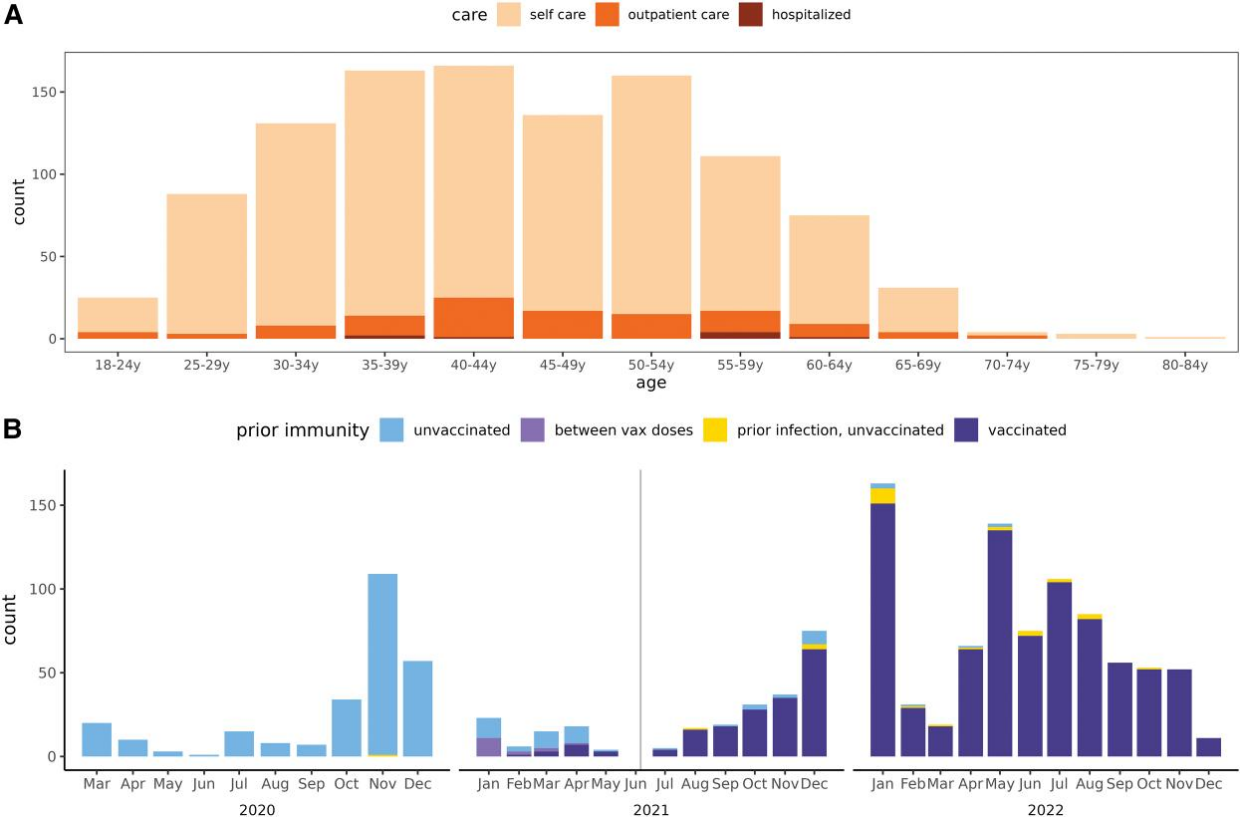
Surveys of IASO (Immunity Associated with SARS-CoV-2) study cases with postacute symptom surveys. *A*, By age and highest level of care sought. *B*, By infection timing and prior immunity status. Note that the care-seeking question was not asked immediately of everyone, so 20% of cases are missing this question.

Between October 2020 and December 2022, 3375 people participated in IASO, contributing a total of 5094 person-years (Supplementary Figure 1). Over that time, 1691 SARS-CoV-2 cases were identified and eligible for a PASC survey (Figure 1). Of these cases, 1370 (81.0%) completed a PASC survey (Figure 1). The age range of cases was 20–82 years, but most were aged 35–55 years (mean age, 44.3 years) and sex was predominantly female (84% female, 16% male); cases that did not have a PASC survey were slightly younger (mean age, 41.5 years) and had a higher percentage of males (19%). The vast majority of IASO cases were self-treated (89%), with only 10% treated in an outpatient setting and 1% hospitalized (Figure 1*A*). Among these cases, 81% (1113/1370) have 2 lines of evidence of infection. Of the 1370 cases with at least 1 PASC survey, 1349 are 3 or more months postinfection and 1232 (91.3%) of these cases have completed their 90-day PASC survey or reported that their symptoms resolved before 90 days (95% of unvaccinated and 90% of vaccinated cases, Supplementary Figure 2).

### SARS-CoV-2 Acute and Postacute Symptoms and Severity

#### Overall

In the acute phase, 93% of cases reported symptoms and 74% reported 5 or more symptoms. At 30 and 90 days postinfection, 38% and 13% of cases reported symptoms, and 6% and 2% reported 5 or more symptoms, respectively (Supplementary Figure 3). The 3 most common symptoms at each time were fatigue (79%), nasal congestion/runny nose (75%), and headache (66%) in the acute phase; fatigue (19%), cough (15%), and cognitive dysfunction (12%) at 30 days; and loss of smell/taste (8%), fatigue (6%), and cognitive dysfunction (5%) at 90 days. Difficulty breathing, the most severe symptom, was reported for 19% of cases in the acute phase, 6% at 30 days, and 2% at 90 days (Supplementary Figure 3). Overall symptom severity did not decrease as noticeably as symptom presence did (mean scores were 2.1, 3.6, and 3.9 for acute phase, 30 days, and 90 days, respectively; Supplementary Figure 4). Thus, while cases were less likely to have symptoms over time, those that remained symptomatic on average did not see a noticeable reduction in their severity (Supplementary Figure 5).

#### Vaccinated Compared to Unvaccinated Cases

By 90 days postinfection, 8% of vaccinated cases were still reporting any symptoms, compared to 27% of unvaccinated cases (Supplementary Figure 6). Any symptoms were 37% and 69% less common for vaccinated cases at 30 and 90 days postinfection (RR, 0.63 [95% confidence interval {CI}, .52–.76] and 0.31 [95% CI, .22–.42]), respectively (Supplementary Figure 6, Supplementary Table 1). Similarly, having 5 or more symptoms was 63% and 66% less common for vaccinated cases at 30 and 90 days postinfection (RR, 0.37 [95% CI, .24–.58] and 0.34 [95% CI, .15–.79]), respectively. There was no difference in the presence of these summary symptoms during the acute phase (Supplementary Figure 6). Vaccinated cases also had significantly lower average and maximum severity scores at all timepoints (Supplementary Figure 7). The average scores for vaccinated cases were lower by 0.70, 2.54, and 2.70 points at the acute phase, 30 days, and 90 days postinfection (relative severity [RS], −0.70 [95% CI, −.51 to −.88]; −2.54 [95% CI, −2.12 to −2.96]; and −2.70 [95% CI, −1.68 to −3.73]), respectively. Maximum severity scores were lower for vaccinated cases by 1.11, 2.26, and 2.07 points at the acute phase, 30 days, and 90 days postinfection (RS, −1.11 [95% CI, −.80 to −1.43]; −2.26 [95% CI, −1.75 to −2.76]; and −2.07 [95% CI, −.75 to −3.38]), respectively.

When we examined specific symptoms, vaccinated cases had more acute upper respiratory symptoms (sore throat: RR, 1.72 [95% CI, 1.42–2.09]; nasal congestion: RR, 1.50, [95% CI, 1.27–1.77]; cough: RR, 1.44 [95% CI, 1.22–1.71]), yet acute upper respiratory symptoms were no more severe. Vaccinated cases had lower severity with less acute and PASC lower respiratory symptoms. Difficulty breathing was 46%, 58%, and 75% less common for vaccinated cases at the acute phase and 30 and 90 days postinfection (RR, 0.54 [95% CI, .42–.70]; 0.28 [95% CI, .18–.44]; and 0.25 [95% CI, .12–.55]), respectively. Other symptoms were also less common for vaccinated cases at 30 and 90 days postinfection, including muscle or body aches, fatigue, chills, sleep issues, loss of smell/taste, headaches, GI issues, and cognitive dysfunction. Even more notably, symptom severity was consistently lower at the acute phase and 30 days postinfection (eg, 30-day difficulty breathing: RS, −3.48 [95% CI, −2.27 to −4.70]).

Interestingly, individual symptom severity trajectories were different for vaccinated and unvaccinated cases (Supplementary Figure 8). Vaccinated cases had clear declines in severity on average (while a small number of vaccinated cases did experience increased severity over time) for many symptoms (eg, cough, fatigue, difficulty breathing), while many unvaccinated cases maintained at the same severity levels over time.

#### Pre-Omicron Vaccinated Compared to Unvaccinated Cases

**Figure 2.**
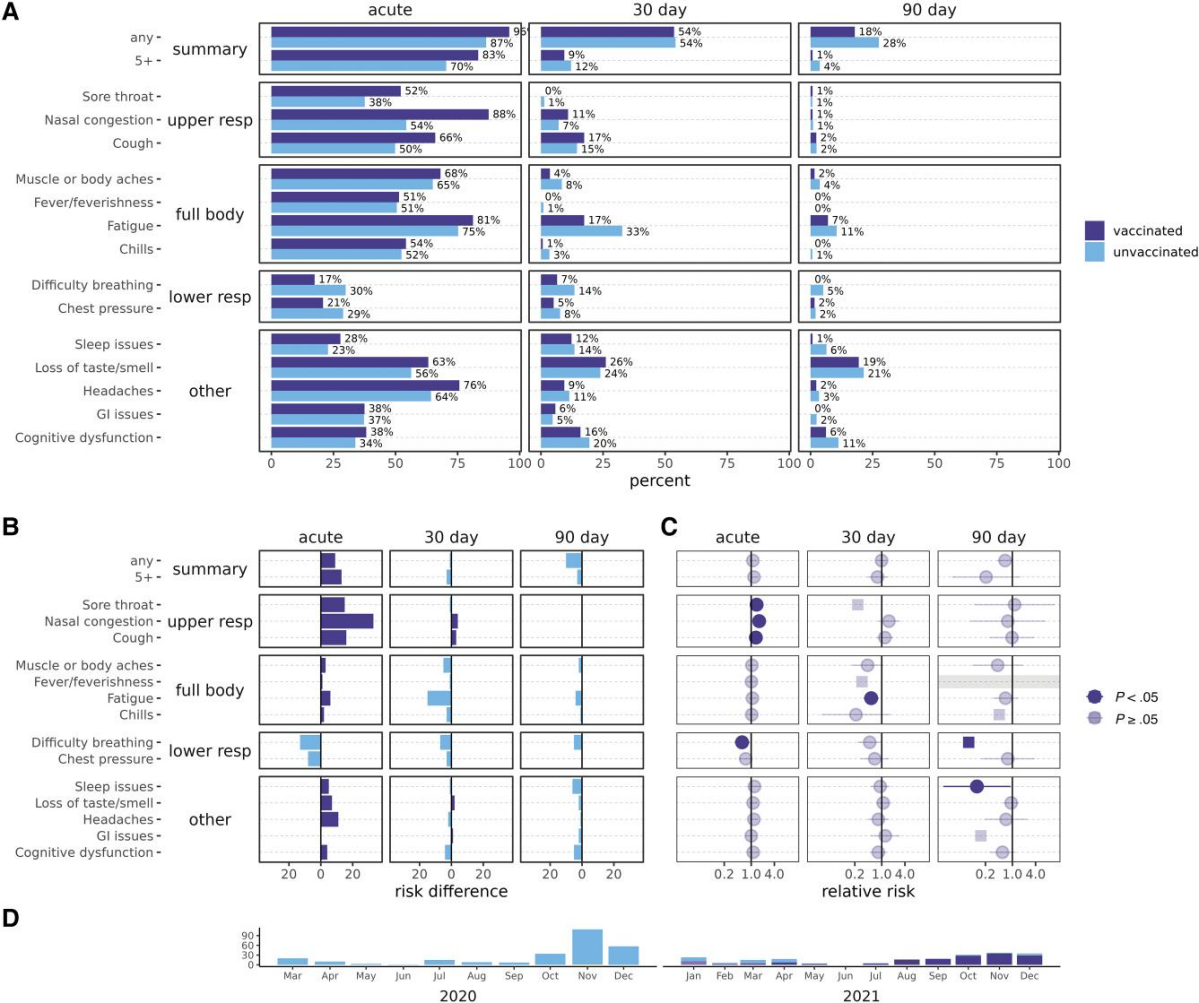
Severe acute respiratory syndrome coronavirus 2 acute and postacute symptom prevalence, among unvaccinated and vaccinated cases, pre-Omicron. Unvaccinated are in light blue/lower and vaccinated in dark blue/upper bars. *A*, Percentages reporting symptoms. *B*, Percentage point difference in reported symptoms, colored by the group reporting more symptoms. *C*, Relative risks for vaccinated vs unvaccinated cases. Squares indicate relative risks with a 0 count of symptoms that were calculated by adding 0.5 to all cells. Grayed-out symptoms indicate comparisons that could not be made due to both groups reporting 0 symptoms. *D*, Plot of the subset of cases over time included in comparisons. Abbreviations: GI, gastrointestinal; resp, respiratory.

**Figure 3.**
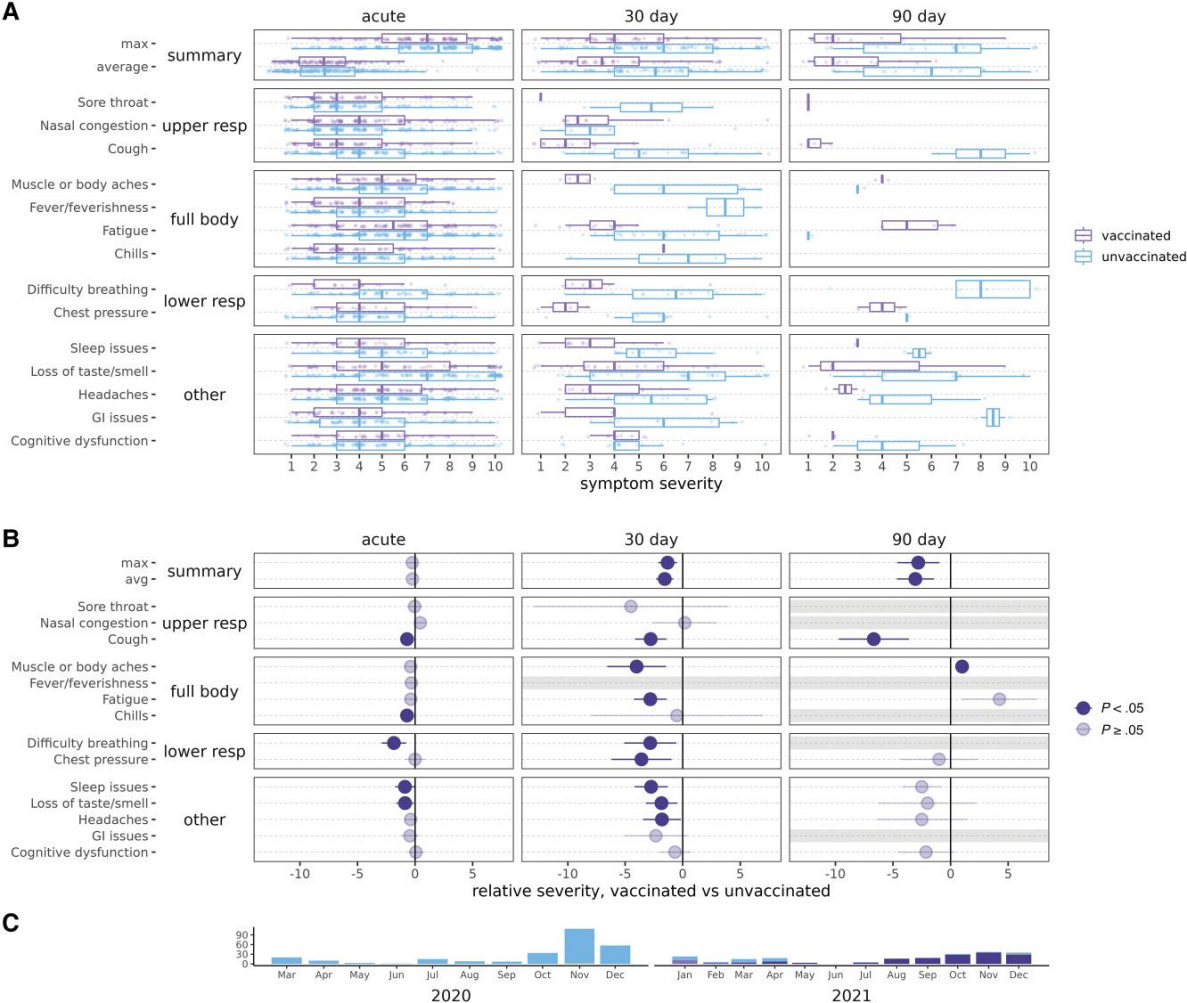
Severe acute respiratory syndrome coronavirus 2 acute and postacute symptom severity, among unvaccinated and vaccinated cases, pre-Omicron. Unvaccinated are in light blue/lower and vaccinated in dark blue/upper boxplots. *A*, Severity of symptoms reported. *B*, Relative severity for vaccinated vs unvaccinated cases, indicating the difference in severity scores on a scale of 1–10. Grayed-out symptoms indicate comparisons that could not be made due to no reported symptom severity for at least 1 group. *C*, Plot of the subset of cases over time included in comparisons. Abbreviations: GI, gastrointestinal; resp, respiratory.

To examine whether differences observed in vaccinated and unvaccinated cases might be due in part to which variant was circulating, additional analyses limited to the pre-Omicron period or vaccinated cases were performed. Pre-Omicron, 18% of vaccinated cases were still reporting any symptoms at 90 days, compared to 28% of unvaccinated cases (Figure 2); however, summary symptoms (any or ≥5) were not significantly lower for vaccinated cases (Figure 2). Pre-Omicron vaccinated cases had lower PASC average and maximum severity scores (eg, at 90 days: average RS, −3.06 [95% CI, −1.44 to −4.67]; maximum RS, −2.81 [95% CI, −.97 to −4.64]) (Figure 3, Supplementary Table 2).

Pre-Omicron, difficulty breathing was lower across at all time points (acute RR, 0.58 [95% CI, .37–.90]; 30-day RR, 0.49 [95% CI, .24–1.00]; 90-day RR, 0.07; *P* = .01, Fisher exact text), acute upper respiratory symptoms were more common, and many symptoms were less severe including difficulty breathing (30-day RS, −2.82 [95% CI, −.56 to −5.08]), but there were fewer significant differences than observed in the overall analysis. Individual symptom severity trajectories were similar to those including all cases (Supplementary Figure 9).

#### Vaccinated Omicron Cases Compared to Vaccinated Pre-Omicron Cases

**Figure 4.**
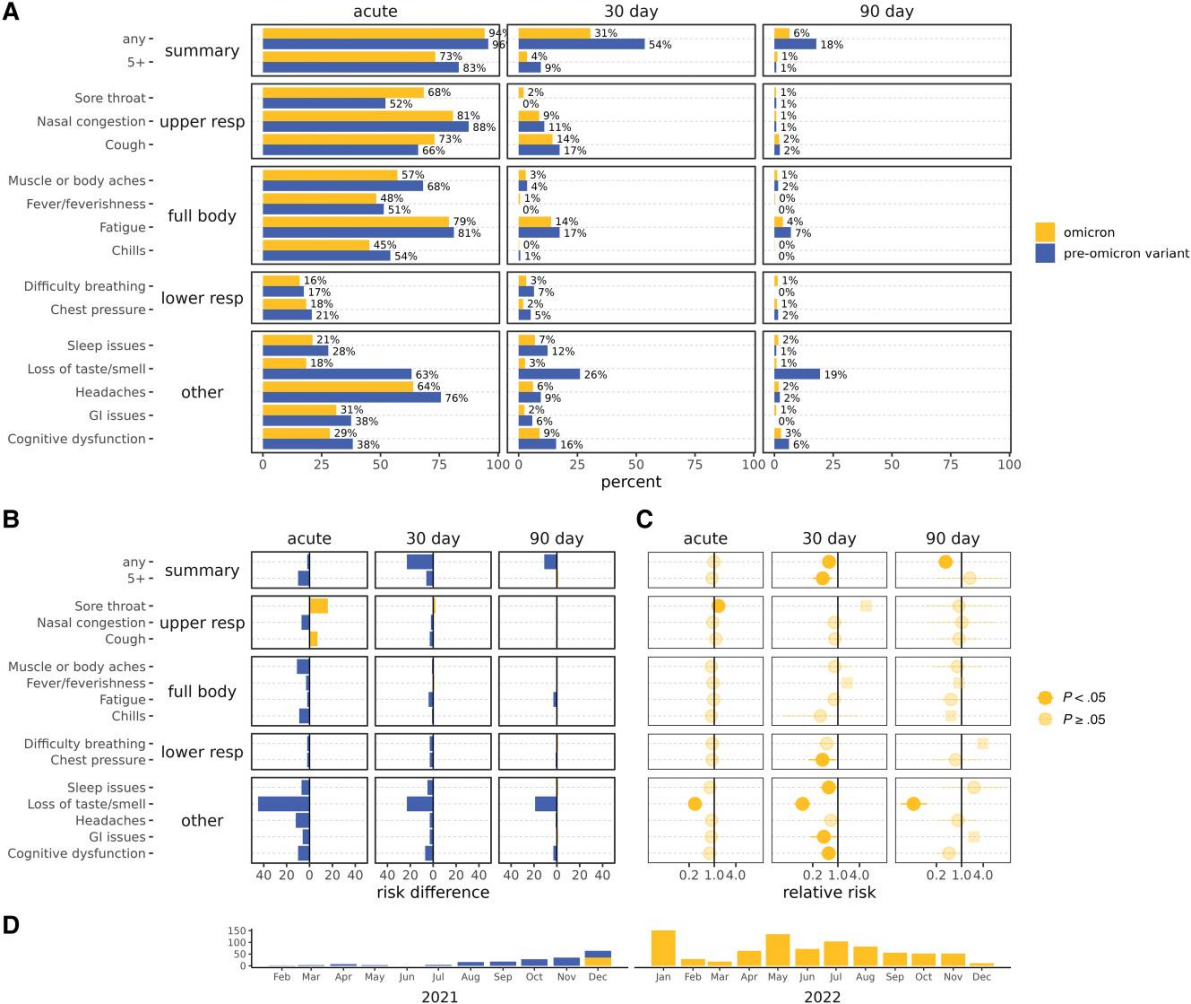
Severe acute respiratory syndrome coronavirus 2 acute and postacute symptom prevalence, among Omicron and pre-Omicron variant vaccinated cases. Omicron is in yellow/upper and pre-Omicron variant in blue/lower bars. *A*, Percentages reporting symptoms. *B*, Percentage point difference in reported symptoms, colored by the group reporting more symptoms. *C*, Relative risks for Omicron vs pre-Omicron variant vaccinated cases. Squares indicate relative risks with 0 counts of symptoms that were calculated by adding 0.5 to all cells. *D*, Plot of the subset of cases over time included in comparisons. Abbreviations: GI, gastrointestinal; resp, respiratory.

**Figure 5.**
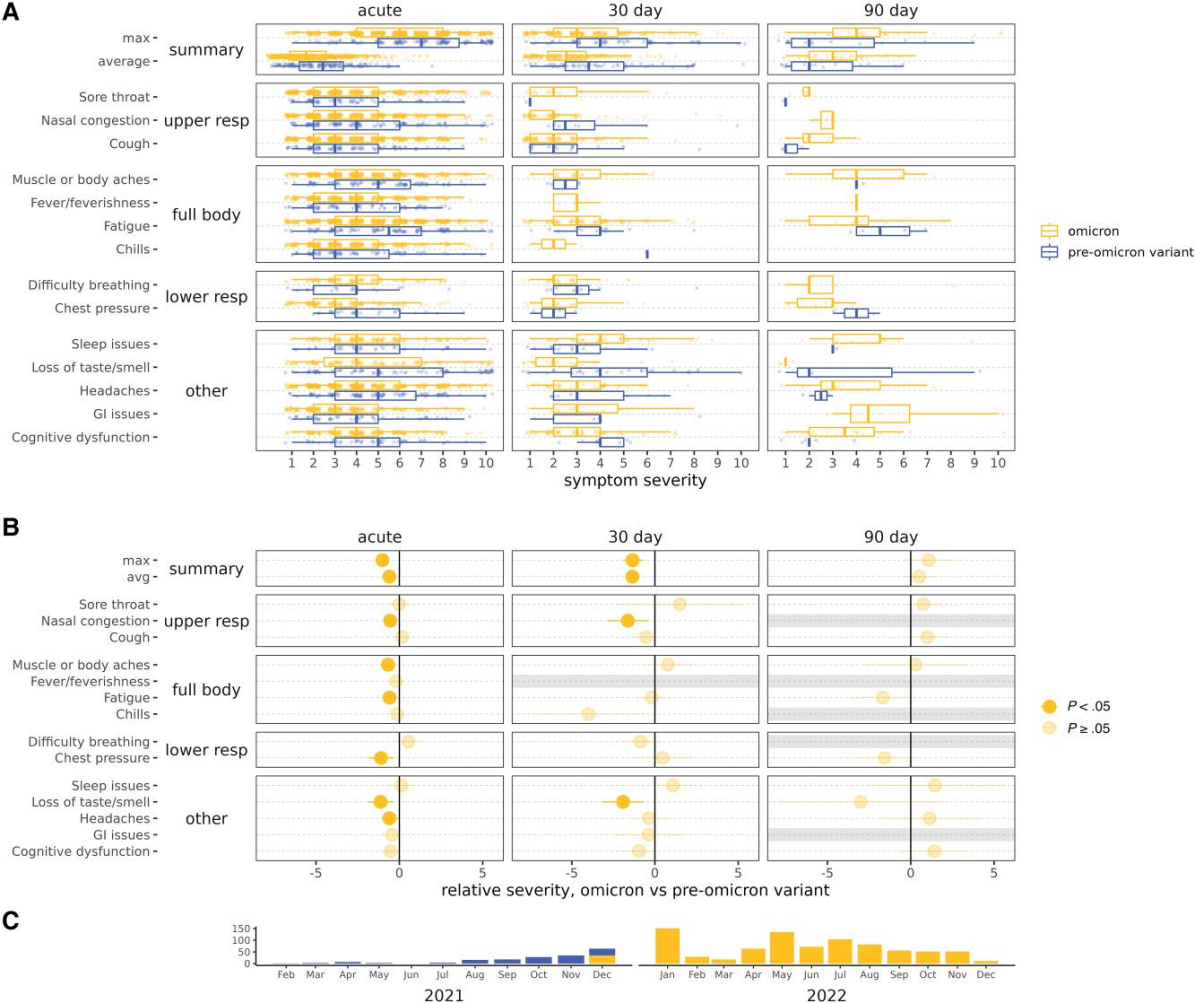
Severe acute respiratory syndrome coronavirus 2 acute and postacute symptom severity, among Omicron and pre-Omicron variant vaccinated cases. Omicron is in yellow/upper and pre-Omicron variant in blue/lower boxplots. *A*, Severity of symptoms reported. The summary is the mean severity for each symptom. *B*, Relative severity for Omicron vs pre-Omicron variant vaccinated cases, indicating the difference in severity scores on a scale of 1–10. Grayed-out symptoms indicate comparisons that could not be made due to no reported symptom severity for at least 1 group. *C*, Plot of the subset of cases over time included in comparisons. Abbreviations: GI, gastrointestinal; resp, respiratory.

Vaccinated Omicron cases reported the fewest symptoms at 90 days—6%, compared to 18% of vaccinated pre-Omicron variant cases. Vaccinated Omicron cases had 43% and 64% less of any symptoms at 30 and 90 days postinfection compared to vaccinated pre-Omicron cases (RR, 0.57 [95% CI, .44–.74] and 0.36 [95% CI, .22–.59]), respectively (Figure 4, Supplementary Table 3), and less of 5 or more symptoms only at 30 days postinfection (RR, 0.38 [95% CI, .20–.73]). No differences were observed in the presence of acute summary symptoms. Symptom severity for vaccinated Omicron compared to pre-Omicron variant cases was also lower by average and maximum scores through 30 days postinfection (eg, acute maximum severity RS, −1.02 [95% CI, −.60 to −1.43]), but apparently higher severity at 90 days postinfection (Figure 5). The individual symptom trajectories (Supplementary Figure 10) help explain the higher severity at 90 days for vaccinated Omicron cases. While symptoms continued declining to nothing by 90 days for many vaccinated Omicron cases, symptoms that did persist often remained at a constant severity. While increases in severity were rare, more vaccinated Omicron than pre-Omicron variant cases had increases in symptom severity over time, and decreases in severity were not as notable after 30 days (Supplementary Figure 10).

Notably, loss of smell/taste was substantially less common and less severe, with vaccinated Omicron cases reporting 71%, 90%, and 95% less at acute phase, 30 days postinfection, and 90 days postinfection (RR, 0.29 [95% CI, .22–.38]; 0.10 [95% CI, .06–.18]; 0.05 [95% CI, .02–.11]), and 1.12 and 1.91 points less severe at acute phase and 30 days postinfection (RS, −1.12 [95% CI, −.35 to −1.89] and −1.91 [95% CI, −.69 to −3.13]), respectively. Several other symptoms were less common (chest pressure, sleep issues, GI issues, and cognitive dysfunction) and less severe (nasal congestion, muscle/body aches, fatigue, chest pressure, and headache) at individual time points. There were not as many differences in the presence or severity of symptoms among vaccinated cases by variant as there were by prior immunity.

#### Vaccinated Second Infections Compared to Vaccinated First Infections

To answer the question of whether subsequent infections in vaccinated individuals will be less severe and less likely to lead to long COVID, we compared second infections to first infections in vaccinated individuals. The average follow-up time for IASO participants was 1.5 years and the average time between first and second infections was 368 days (range, 61–879 days). Unfortunately, there was no reduction in symptom prevalence or severity of second infections in vaccinated individuals (Supplementary Figures 11 and 12, Supplementary Table 4).

#### Sensitivity Analysis Excluding Acute Asymptomatic Cases

Very few cases (96 cases [7%]) were asymptomatic in the acute phase. We did a sensitivity analysis excluding cases that were asymptomatic in the acute phase, and our conclusions held (Supplementary Figure 1^3^).

## DISCUSSION

We found that overall, 13% of participants still reported symptoms at 90 days postinfection. This is in the range of what others have estimated and on the lower side [1, 19], likely because the IASO population is community-based and very highly vaccinated. The proportion still reporting symptoms at 90 days was lower for vaccinated cases (8%) and lowest for vaccinated Omicron cases (6%).

Vaccination was associated with less acute and PASC symptoms and symptom severity. Other studies have found widely ranging estimates of the effect of vaccination on long COVID symptoms, with odds ratios of 0.2–2.0 [10, 14]. Our estimate of vaccinated cases experiencing 69% less symptoms at 90 days (all cases subset) is lower than some studies, likely due to the fact that our cases are community-based and thus are representative of COVID infections in general, rather than a subset of more severe COVID cases or high-risk populations. Our estimates may therefore better represent the long COVID in the general US working-age adult population. In addition, existing studies used many different definitions of long COVID and most did not report symptom-specific associations. While the overall prevalence of long COVID is important, the impact of specific symptoms on quality of life varies greatly. We found protection from difficulty breathing, even in the pre-Omicron subset where most vaccinated cases were the more severe Delta variant [5]. However, we did not see a reduction in the prevalence of less concerning upper respiratory symptoms. The Omicron variant was most notably associated with less and less severe loss of smell/taste, as others have also found [20].

Because of differences in timing between our vaccinated and unvaccinated cases and differences in severity of circulating variants, in addition to overall prevalence we compared vaccinated and unvaccinated cases in the pre-Omicron era. While this analysis had less power, a clear reduction in severity was seen. This is in spite of the fact that, due to the high vaccine effectiveness against the original strain and early variants, IASO pre-Omicron vaccinated cases occurred mostly in the second half of 2021, when the more severe Gamma and Delta variants circulated [5, 21]. This suggests that there could be even stronger associations with vaccination and long COVID for less severe variants.

To date, overall, the Omicron variant is less severe during acute illness than prior variants. When we compared infections in vaccinated individuals in the Omicron era to the pre-Omicron era, we found a reduction in long COVID prevalence and severity for Omicron. Thus, the prevalence of long COVID in studies conducted prior to the introduction of Omicron likely overestimates the rates from new infections that would be seen in those populations currently. While we did see a reduction in severity of long COVID with Omicron, it was notable that we did not see a reduction in severity of long COVID in second infections in vaccinated individuals. As the virus mutates and the population immune history evolves, it will be important to monitor changes in long COVID patterns.

The CDC now also recognizes additional common long COVID symptoms such as anxiety, depression, and changes in menstruation. We designed our long COVID survey early in the pandemic and it was finalized in early 2021 when less was known about long COVID, so unfortunately these symptoms were not included in our survey. Importantly, there are many other symptoms and conditions (such as myalgic encephalomyelitis/chronic fatigue syndrome, postural orthostatic tachycardia syndrome, and many others) that have been associated with SARS-CoV-2 [22, 23]. One limitation of our study was that, as a community-based cohort, it was not powered to look at these sorts of rare, severe long COVID outcomes. However, the design is also a strength in that it allows us to capture cases that do not seek out healthcare and thus better estimate the proportion of COVID-19 cases that experience long COVID.

Another limitation is for the vaccinated first compared to second infection comparison: Because the average participant follow-up time was 1.5 years and hybrid immunity provides relatively strong protection, the follow-up time was not sufficient to detect a large number of second infections. Our population is a working, reasonably healthy, mostly middle-aged, and predominantly female population. Results are generalizable to a large part of the general population and do not generalize to children and high-risk populations. Recall bias is also a concern for our study, since we sent long COVID surveys to participants infected before June 2021 retroactively. However, individuals tend to have a fairly good memory of their first COVID illness and the time period was not very long. Also, given that most of the cases surveyed retrospectively were unvaccinated individuals and we would assume an underreporting of symptoms for those who had to recall prior symptoms, we expect that the protective associations we found for vaccination would be even larger in the absence of recall bias.

In summary, we found that approximately 18% of vaccinated cases in the pre-Omicron era and 6% in the Omicron era experienced long COVID symptoms at 90 days postinfection, compared to 27% of unvaccinated cases. Vaccinated individuals were less likely to experience long COVID and have lower long COVID severity than unvaccinated individuals.

## Supplementary Material

ofae039_Supplementary_Data

## Supplementary Data

Supplementary materials are available at *Open Forum Infectious Diseases* online. Consisting of data provided by the authors to benefit the reader, the posted materials are not copyedited and are the sole responsibility of the authors, so questions or comments should be addressed to the corresponding author.

***Acknowledgments*.** We thank all of the IASO participants, and the rest of the team involved in running the IASO study. Gordon group laboratory staff: May Patel, Nivea Vydiswaran, Dan Raymond, Gabe Simjanovski, Mahboob Chowdury, Casey Juntila, Jamie Bird, Wolf Gremel, Julio Zuniga Moya; faculty and administrative staff: John Kubale, Brad Carlson; IASO research assistants: Stephanie Lai, Janay Patterson, Marissa Olsen, Scarlet Cockell, Shriya Yatnalkar, Rebecca Rush, Dylan Landau, Anna Prushinskaya, Jenna Kiryakos, Susan Carpenter, Donna DiFranco, Nikita Patel, Mia Zhu, Kayla Janevski. We also thank the University of Michigan Medical School Central biorepository for providing biospecimen storage, management, and distribution services in support of the research reported in this publication.

***Author contributions.*** H. E. M., T. K.-D., A. E., and A. G. had full access to all the data in the study and take responsibility for the integrity of the data and the accuracy of the data analysis. Concept and design: C. G., D. M., E. S., R. V., and A. G. Methodology for IASO: A. G. Acquired funding: C. G., R. V., and A. G. Provided resources: E. S., R. V., and A. G. Investigation: T. K.-D., C. G., D. M., D. D., K. B., L. W., M. A., A. B., E. S., R. V., and A. G. Supervision: T. K.-D., C. G., D. M., K. B., E. S., R. V., and A. G. Project administration: T. K.-D., C. G., D. M., A. M., L. W., E. S., R. V., and A. G. Data curation and validation: H. E. M., T. K.-D., A. E., A. M., D. D., K. B., K. L., Z. C., L. W. Statistical analysis and visualizations: H. E. M. Original draft: H. E. M. Review and editing: all authors.

***Financial support*.** This work is part of the PARIS/SPARTA studies supported by the National Institute of Allergy and Infectious Diseases Collaborative Influenza Vaccine Innovation Centers (contract 75N93019C00051 to A. G. and R. V.) and is supported by a grant from Open Philanthropy, the University of Michigan Center for Infectious Disease Threats and Office of the Vice President for Research. A. G. is supported by the Biosciences Initiative at the University of Michigan through a Mid-career Biosciences Faculty Achievement Award.
