# Supplementary material for: Reduction in Long COVID Symptoms and Symptom Severity in Vaccinated Compared to Unvaccinated Adults
Source: Open Forum Infect Dis. 2024 Jan 23;11(2):ofae039. doi: 10.1093/ofid/ofae039 (PMC10847810; doi:10.1093/ofid/ofae039)
Supplement: ofae039_Supplementary_Data [file ofae039_supplementary_data.docx]

Supplement for

Reduction in long-COVID symptoms and symptom severity in vaccinated compared to unvaccinated adults

Table of contents

Figures:

1. Study flowchart
2. River plot of acute and post-acute sequelae of COVID (PASC) results
3. Acute and PASC symptom prevalence, overall
4. Acute and PASC symptom severity, overall
5. Individual symptom severity trajectories, overall
6. Acute and PASC symptom prevalence, by prior immunity, among all cases
7. Acute and PASC symptom severity, by prior immunity, among all cases
8. Individual symptom severity trajectories, by prior immunity, among all cases
9. Individual symptom severity trajectories, by prior immunity, among pre-Omicron cases
10. Individual symptom severity trajectories, by variant, among vaccinated cases
11. Acute and PASC symptom prevalence, by prior infection status among vaccinated cases
12. Acute and PASC symptom severity, by prior infection status among vaccinated cases
13. Sensitivity analysis excluding acute asymptomatic cases for: Acute and PASC symptom prevalence, by prior immunity, among all cases

Tables:

1. Symptom prevalence ratios for vaccinated compared to unvaccinated among all cases
2. Symptom prevalence ratios for vaccinated compared to unvaccinated among pre-omicron cases
3. Symptom prevalence ratios for Omicron compared to pre-Omicron variant among vaccinated cases
4. Symptom prevalence ratios for prior infected compared to prior uninfected among vaccinated cases


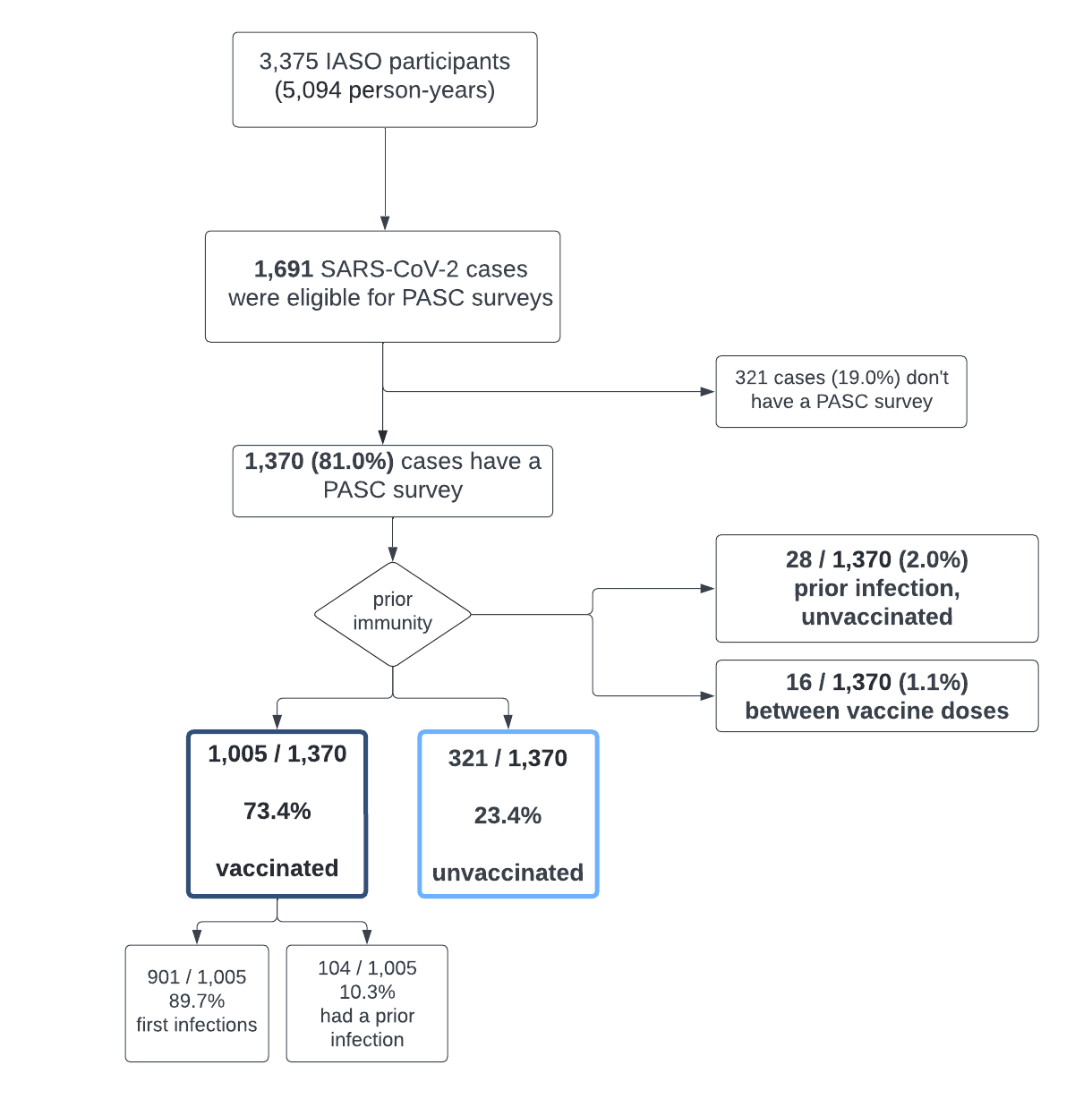


**Figure S1. Study flowchart.**


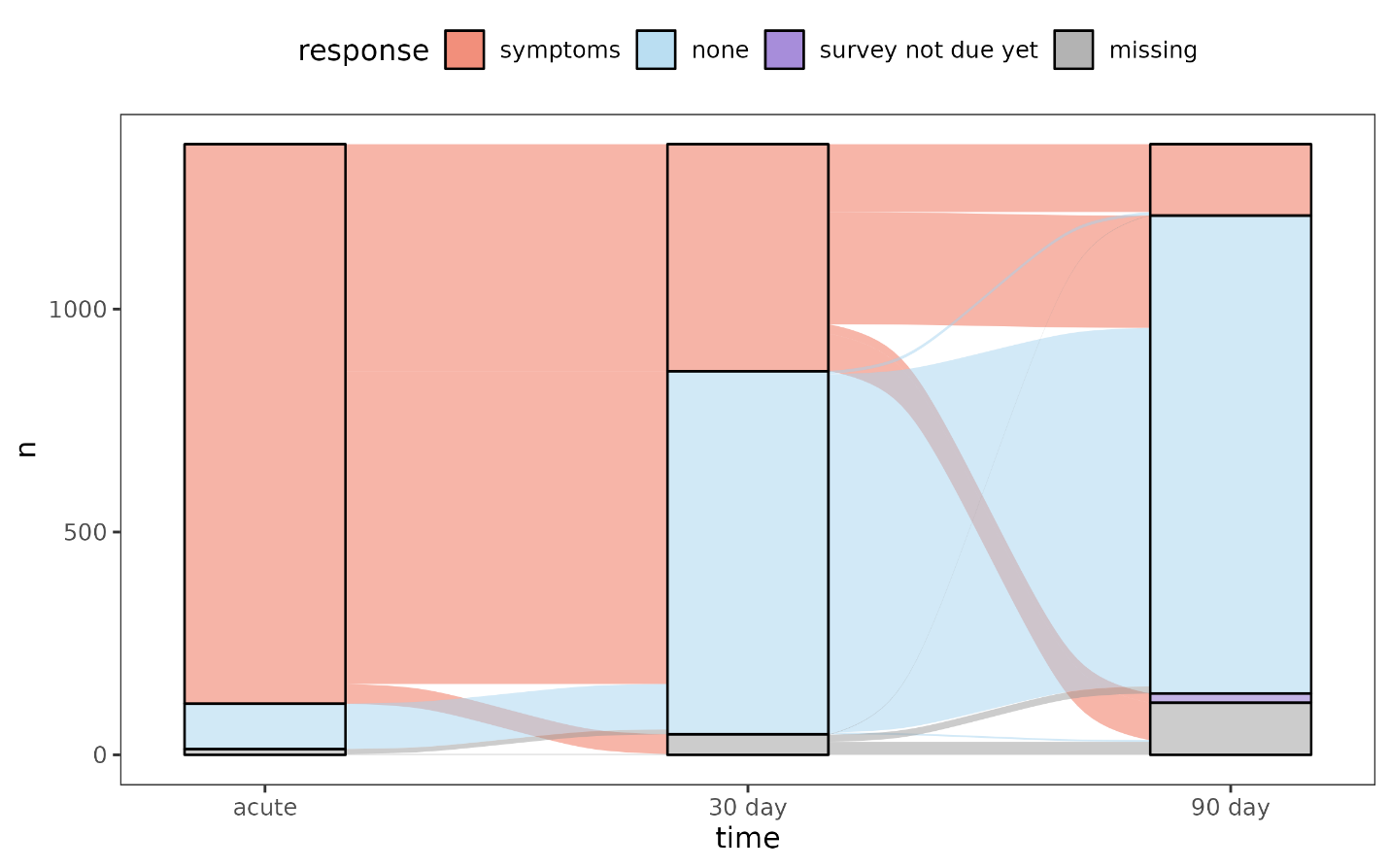


**Figure S2. River plot of acute and post-acute sequelae of COVID (PASC) results.** We are watching the continuation of symptoms, not the development of new or different symptoms.


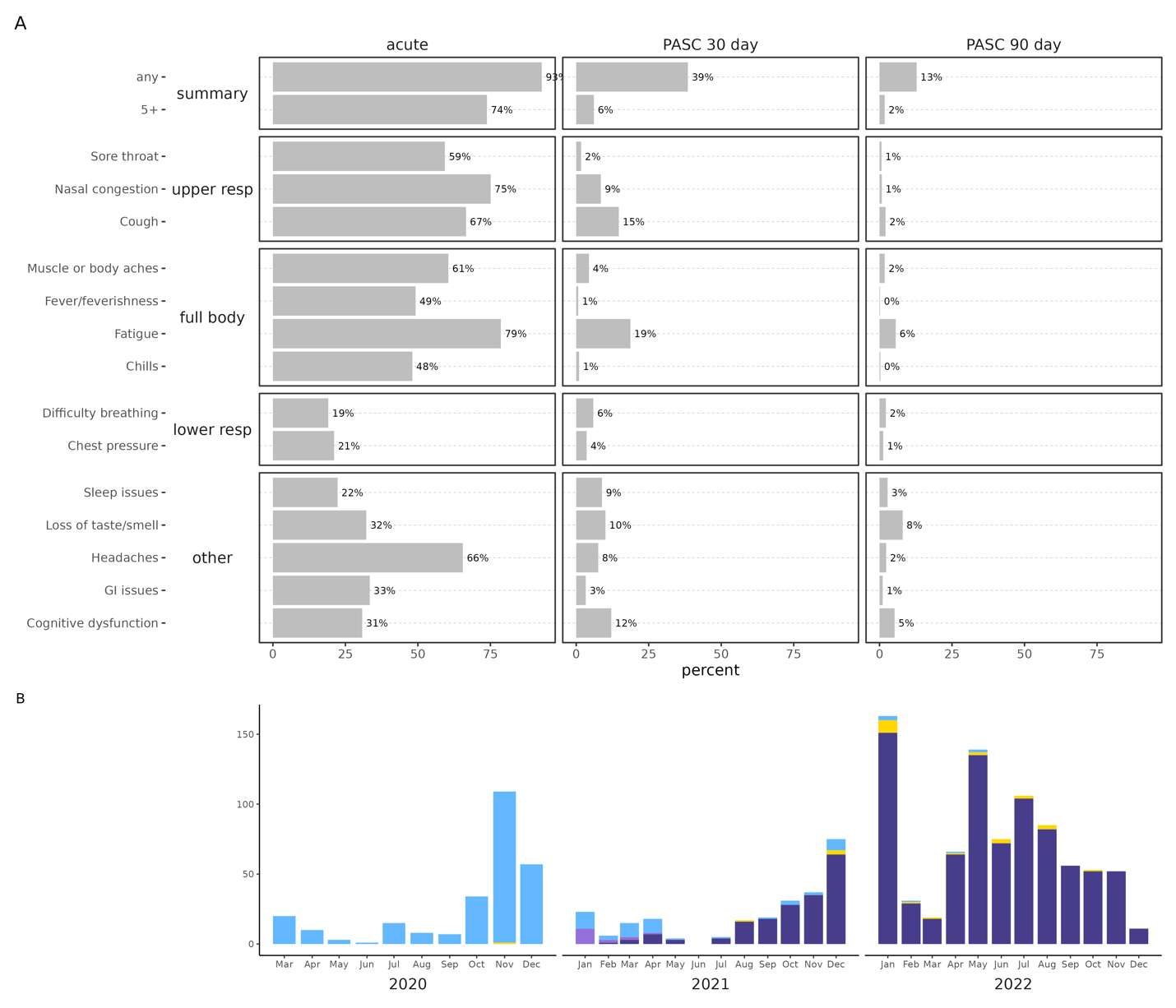


**Figure S3. SARS-CoV-2 acute and post-acute (PASC) symptom prevalence, overall.** A) Percentages reporting symptoms. B) Plot of the subset of cases over time included.


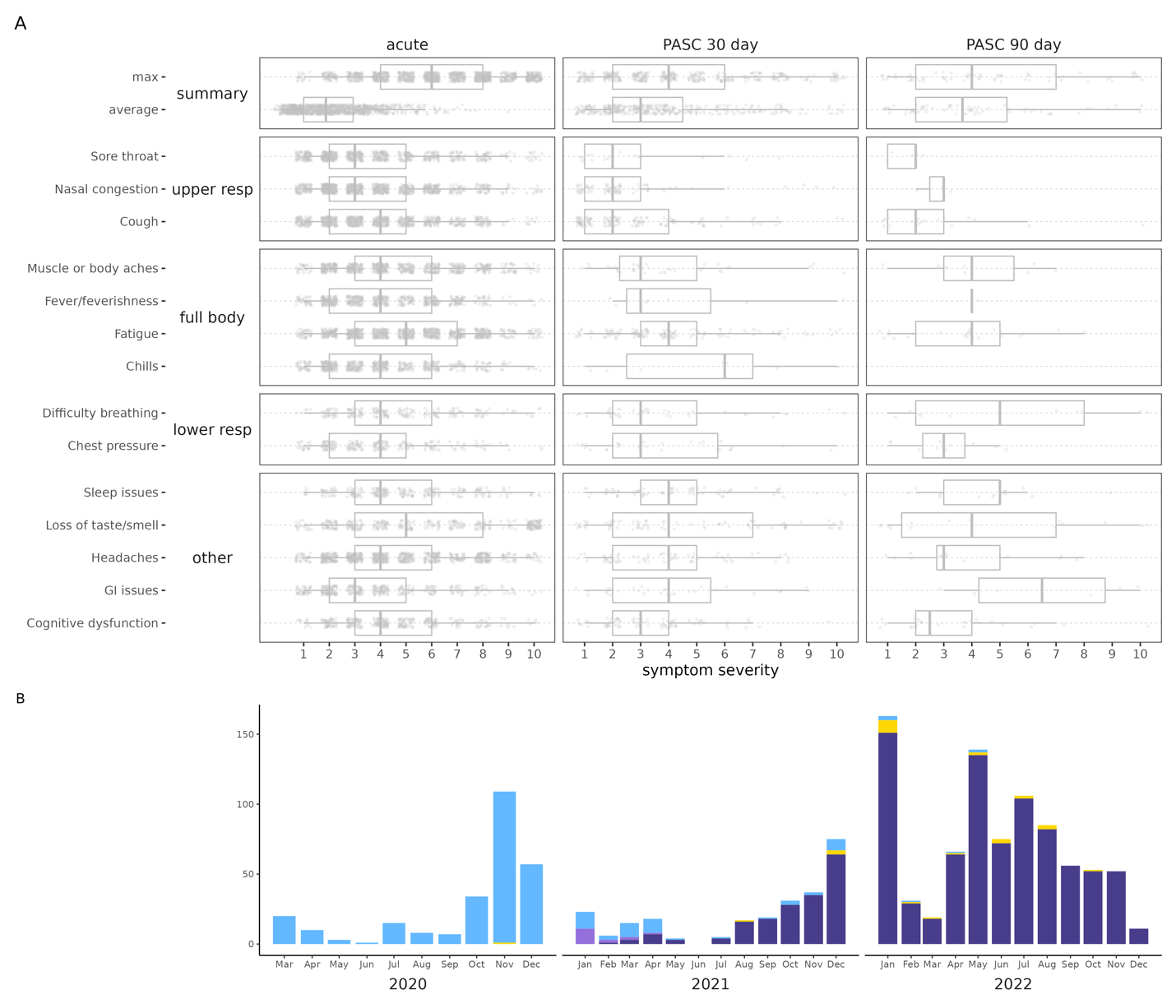


**Figure S4. SARS-CoV-2 acute and post-acute (PASC) symptom severity, overall. A**) Percentages reporting symptoms. B) Plot of the subset of cases over time included.


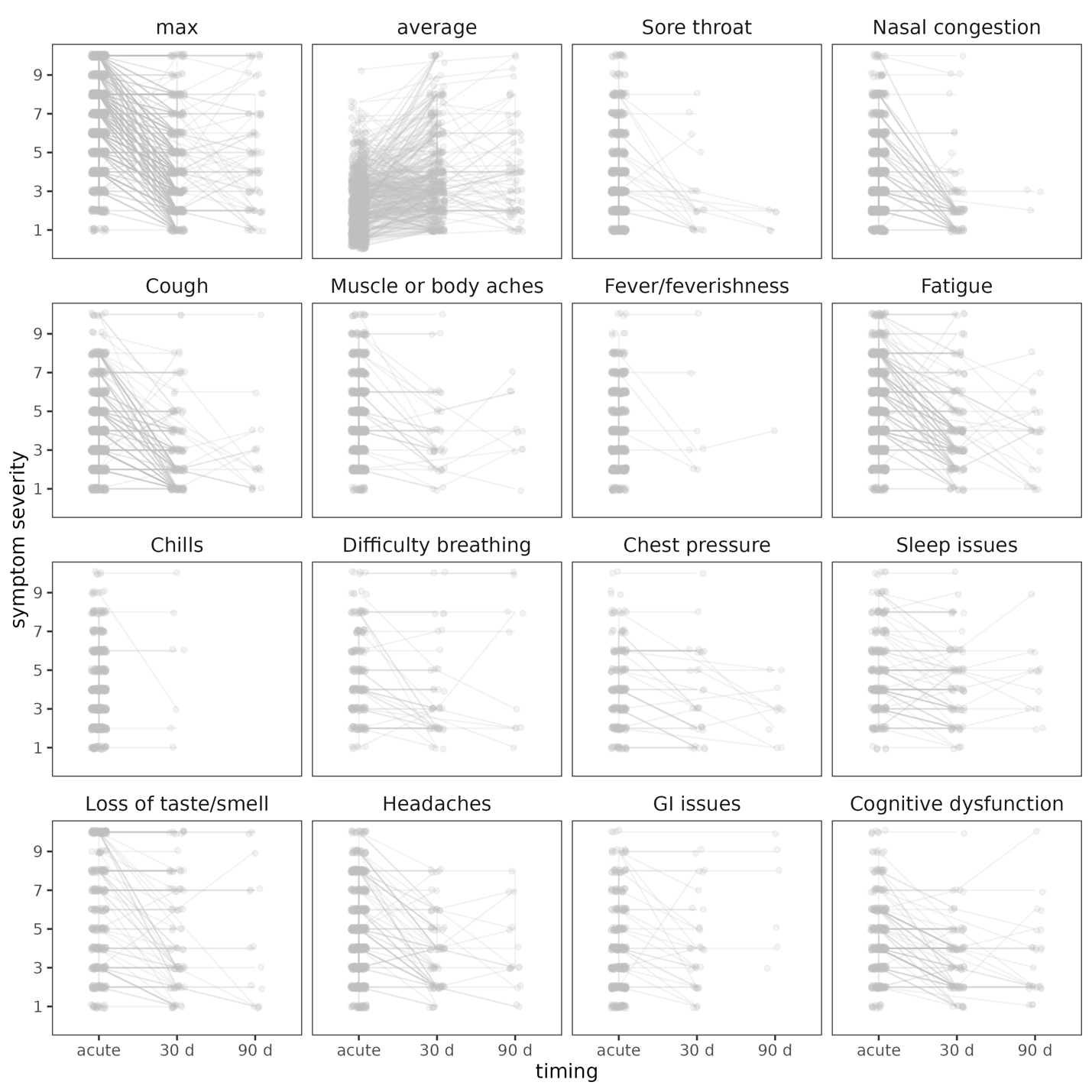


**Figure S5. Individual symptom severity trajectories, overall.**


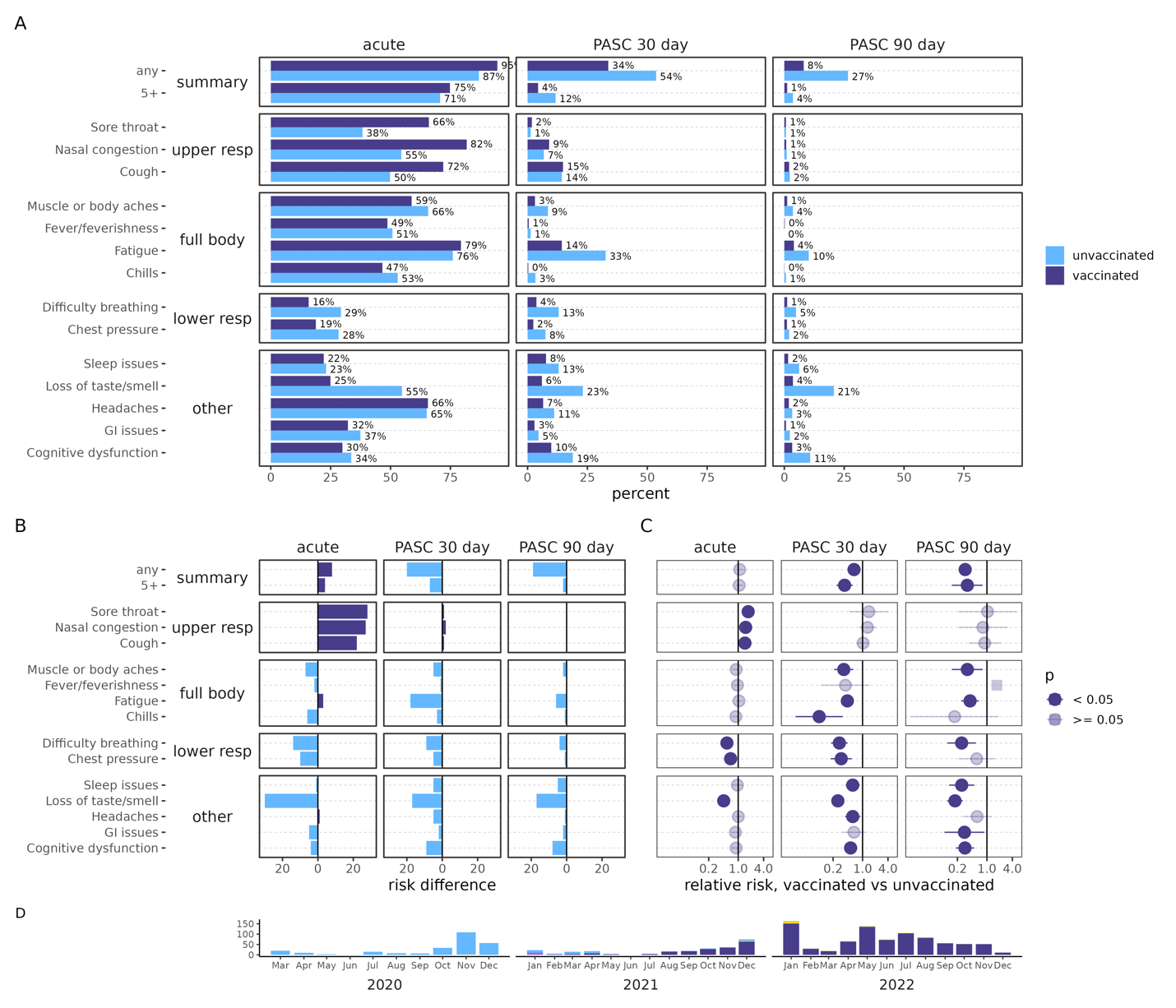


**Figure S6. Acute and PASC symptom prevalence, by prior immunity, among all cases.** Unvaccinated are in light blue and vaccinated in dark blue. A) Percentages reporting symptoms. B) Percentage point difference in reported symptoms, colored by the group reporting more symptoms. C) Relative risks for vaccinated vs unvaccinated cases. Squares indicate relative risks with a 0 count of symptoms that were calculated by adding 0.5 to all cells. Greyed out symptoms indicate comparisons that could not be made due to both groups reporting 0 symptoms. D) Plot of the subset of cases over time included in comparisons.


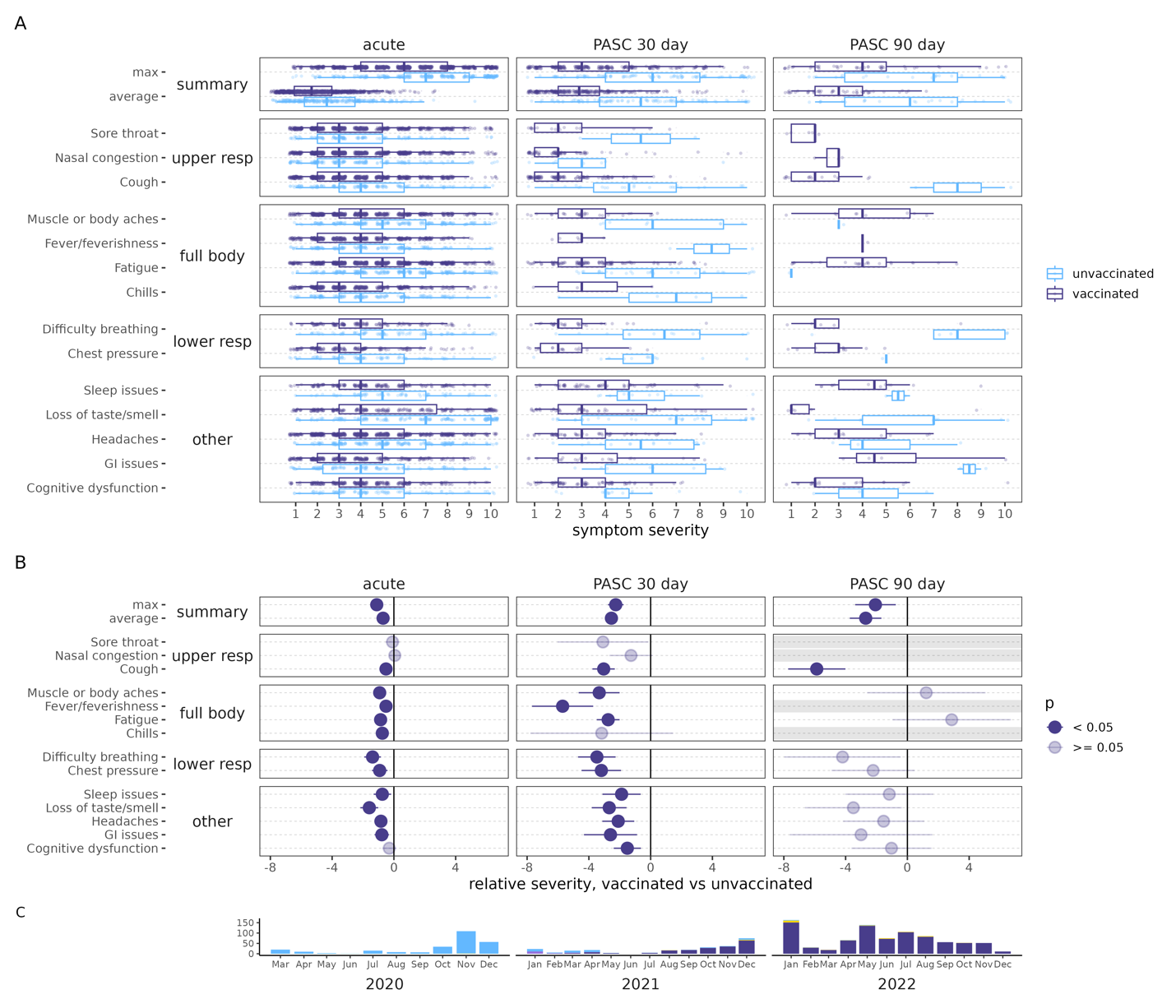


**Figure S7. Acute and PASC symptom severity, by prior immunity, among all cases.** Unvaccinated are in light blue and vaccinated in dark blue. A) Severity of symptoms reported. B) Relative severity for vaccinated vs unvaccinated cases, indicating the difference in severity scores on a scale of 1-10. Greyed out symptoms indicate comparisons that could not be made due to no reported symptom severity for at least one group. C) Plot of the subset of cases over time included in comparisons.


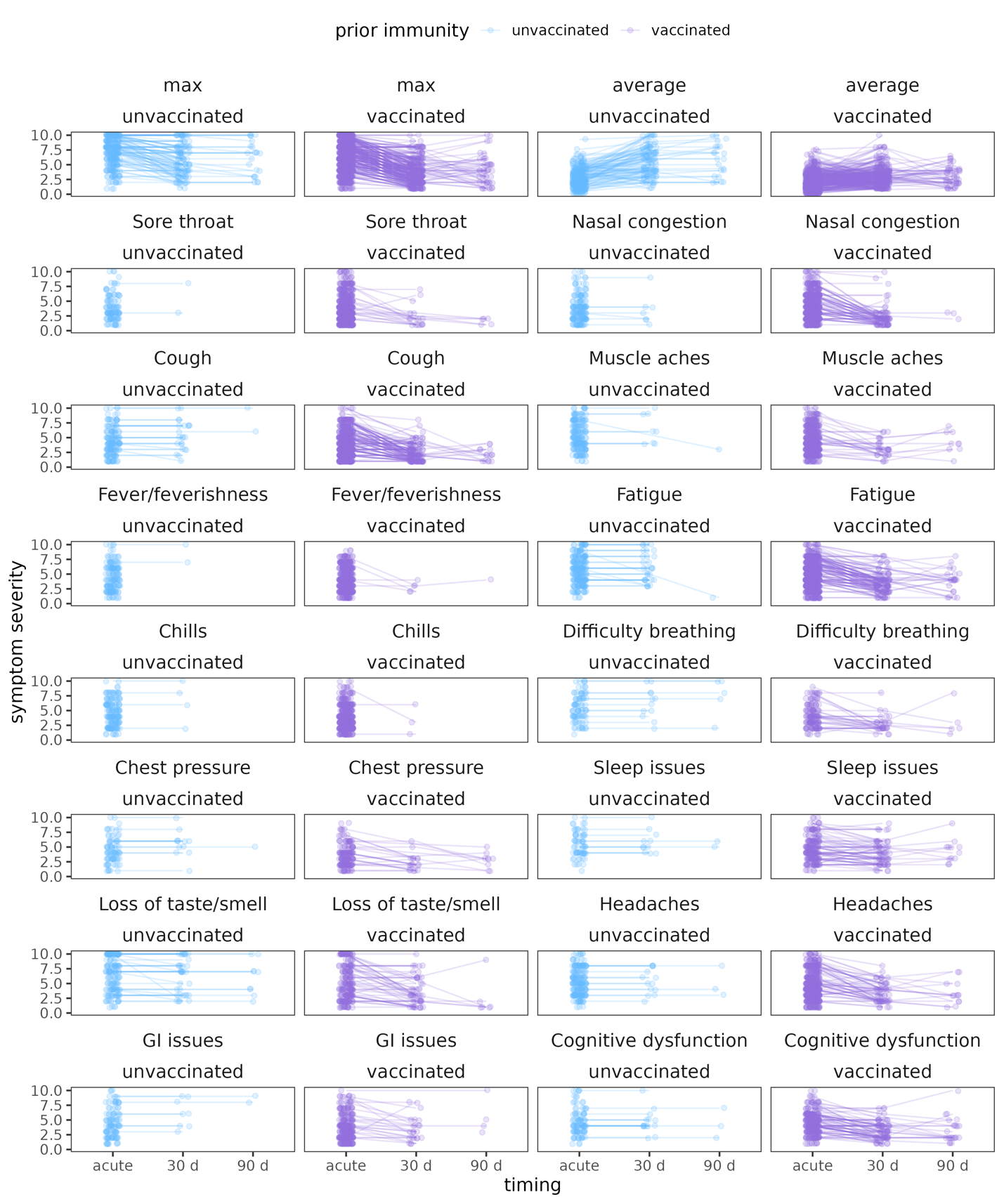


**Figure S8. Individual symptom severity trajectories, by prior immunity, among all cases.**


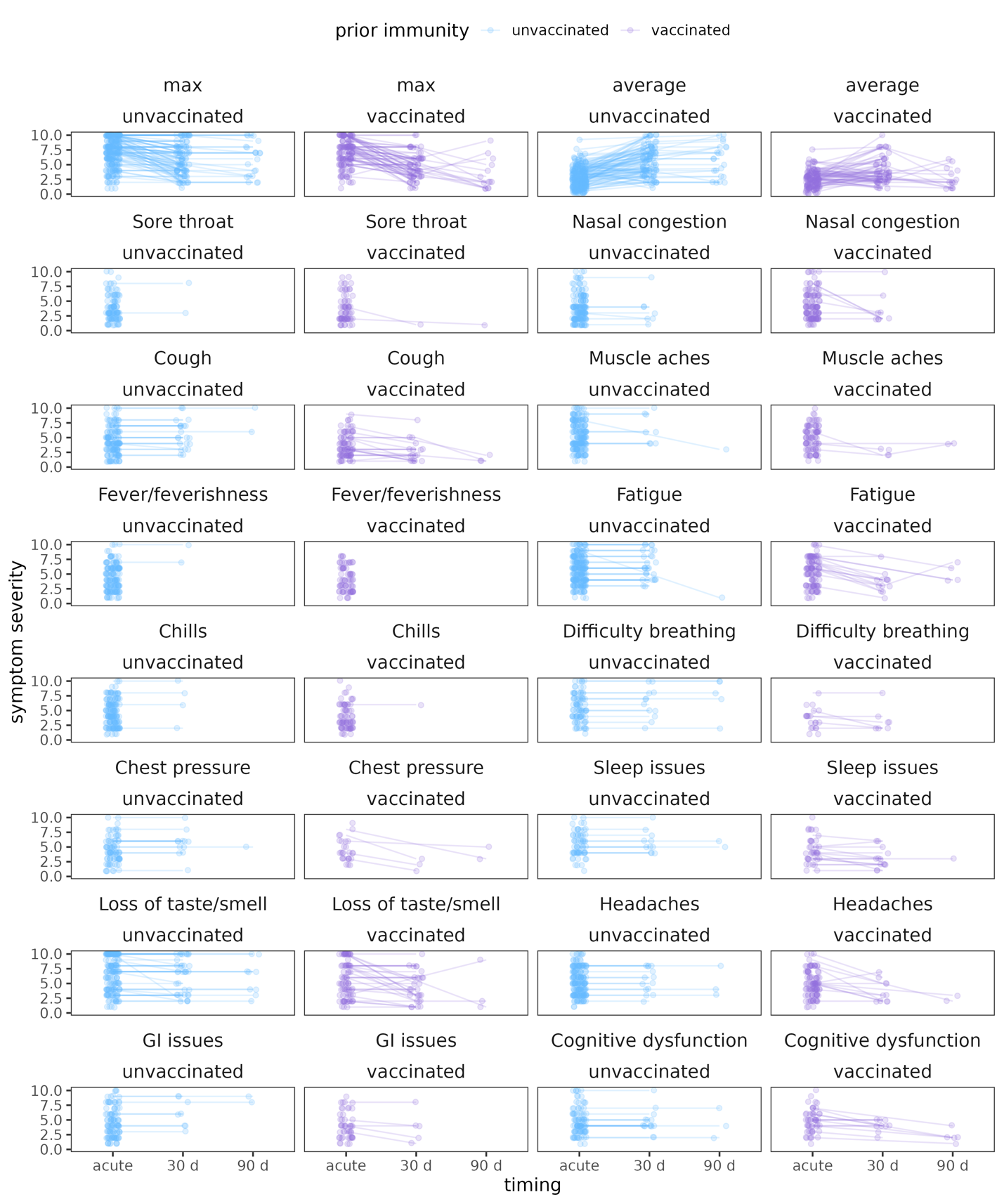


**Figure S9. Individual symptom severity trajectories, by prior immunity, among pre-Omicron cases.**


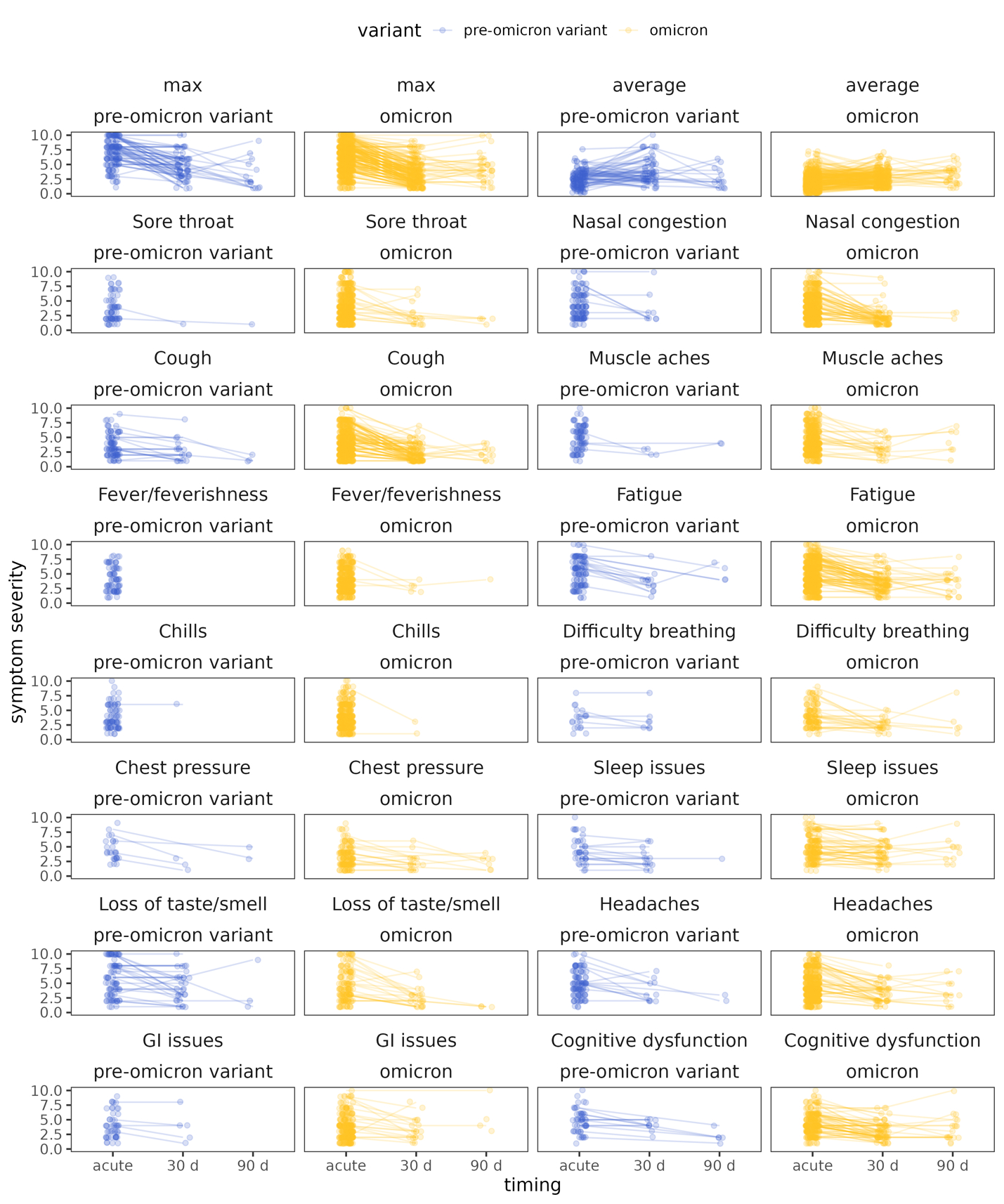


Figure S10. Individual symptom severity trajectories, by variant, among vaccinated cases.


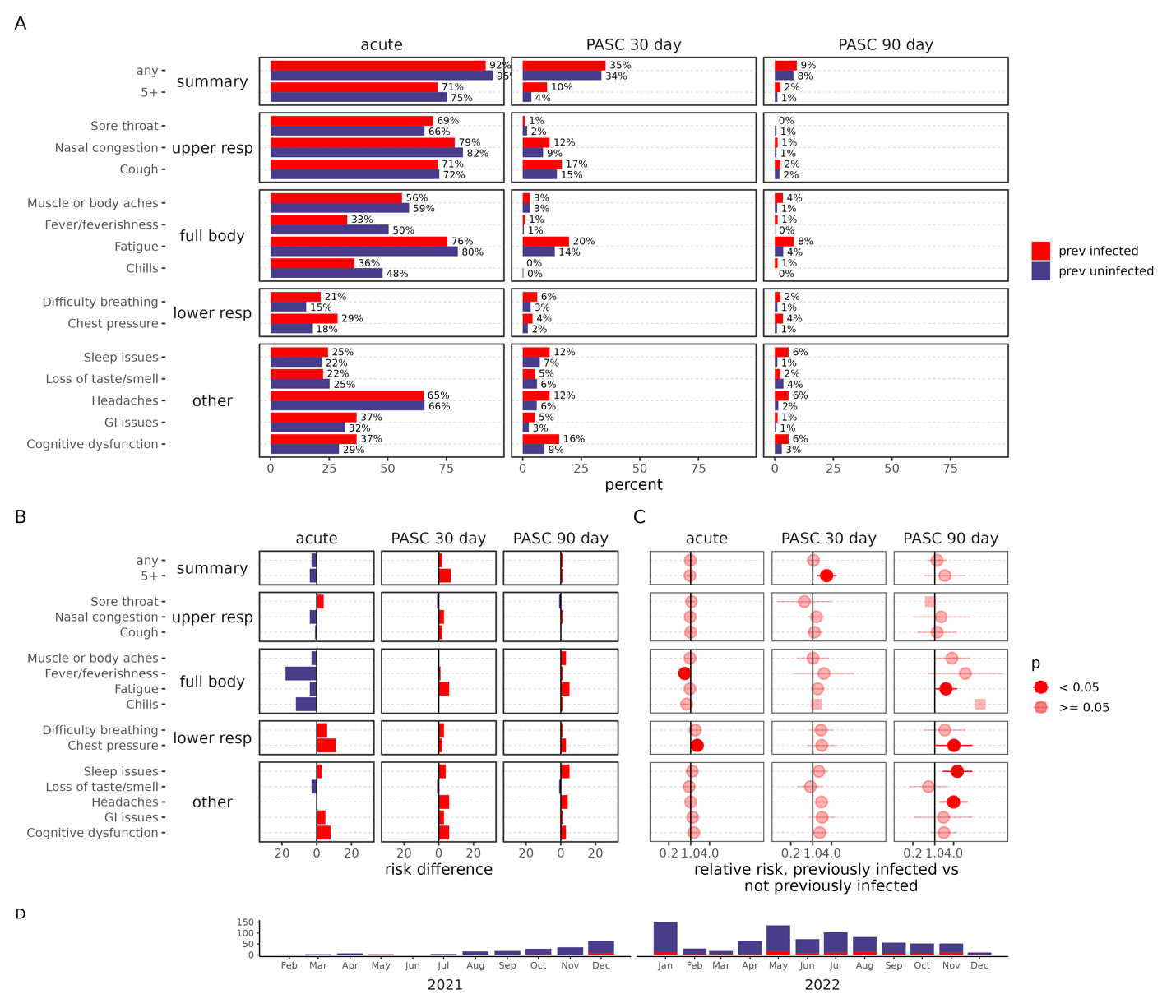


**Figure S11. Acute and PASC symptom prevalence, by prior infection status among vaccinated cases.** Previously infected is in red and not previously infected in purple. A) Percentages reporting symptoms. B) Percentage point difference in reported symptoms, colored by the group reporting more symptoms. C) Relative risks for previously infected vs not previously infected. Squares indicate relative risks with a 0 count of symptoms that were calculated by adding 0.5 to all cells. D) Plot of the subset of cases over time included in comparisons.


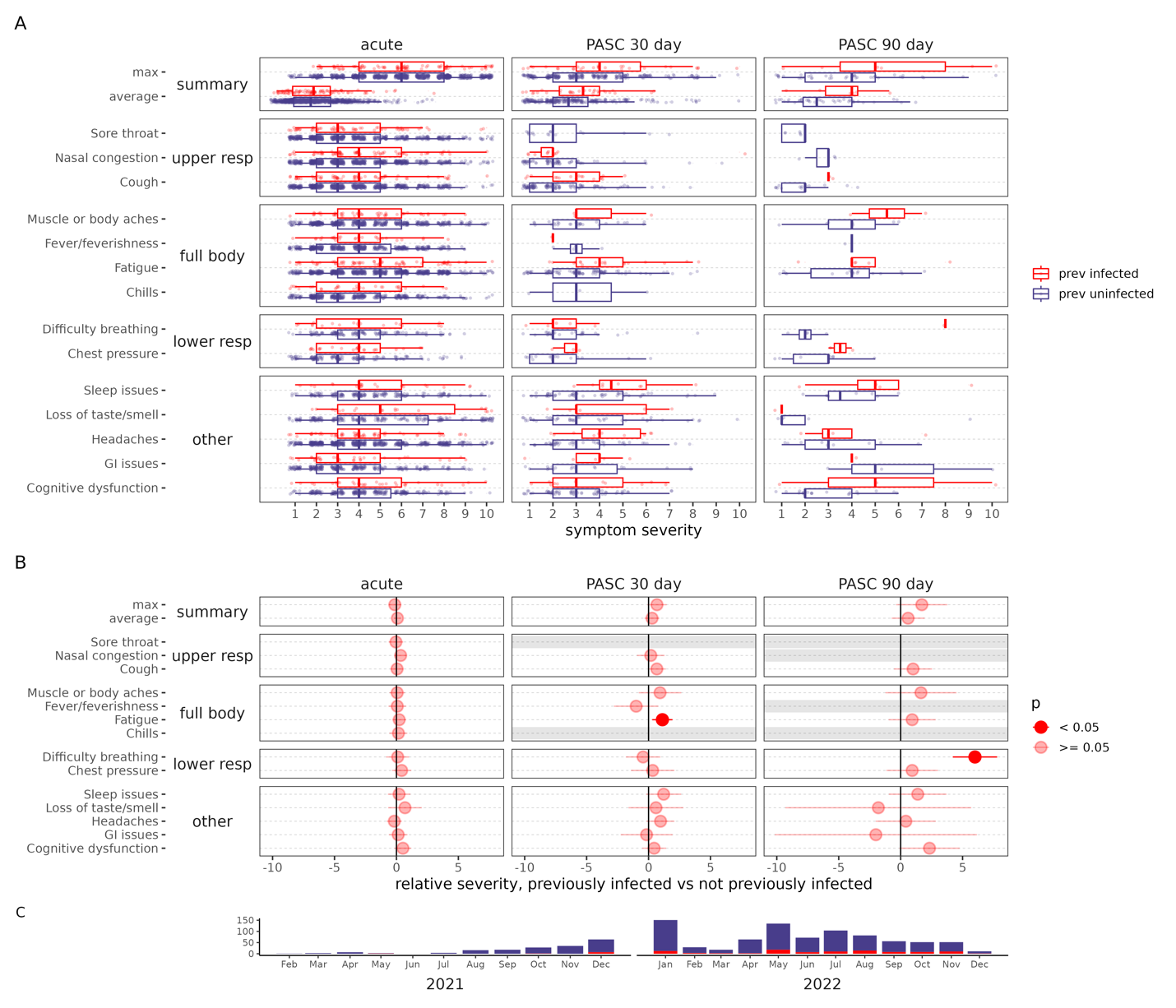


**Figure S12 Acute and PASC symptom severity, by prior infection status among vaccinated cases.** Previously infected is in red and not previously infected in purple. A) Severity of symptoms reported. B) Relative severity for previously infected vs not previously infected, indicating the difference in severity scores on a scale of 1-10. Greyed out symptoms indicate comparisons that could not be made due to no reported symptom severity for at least one group. C) Plot of the subset of cases over time included in comparisons.


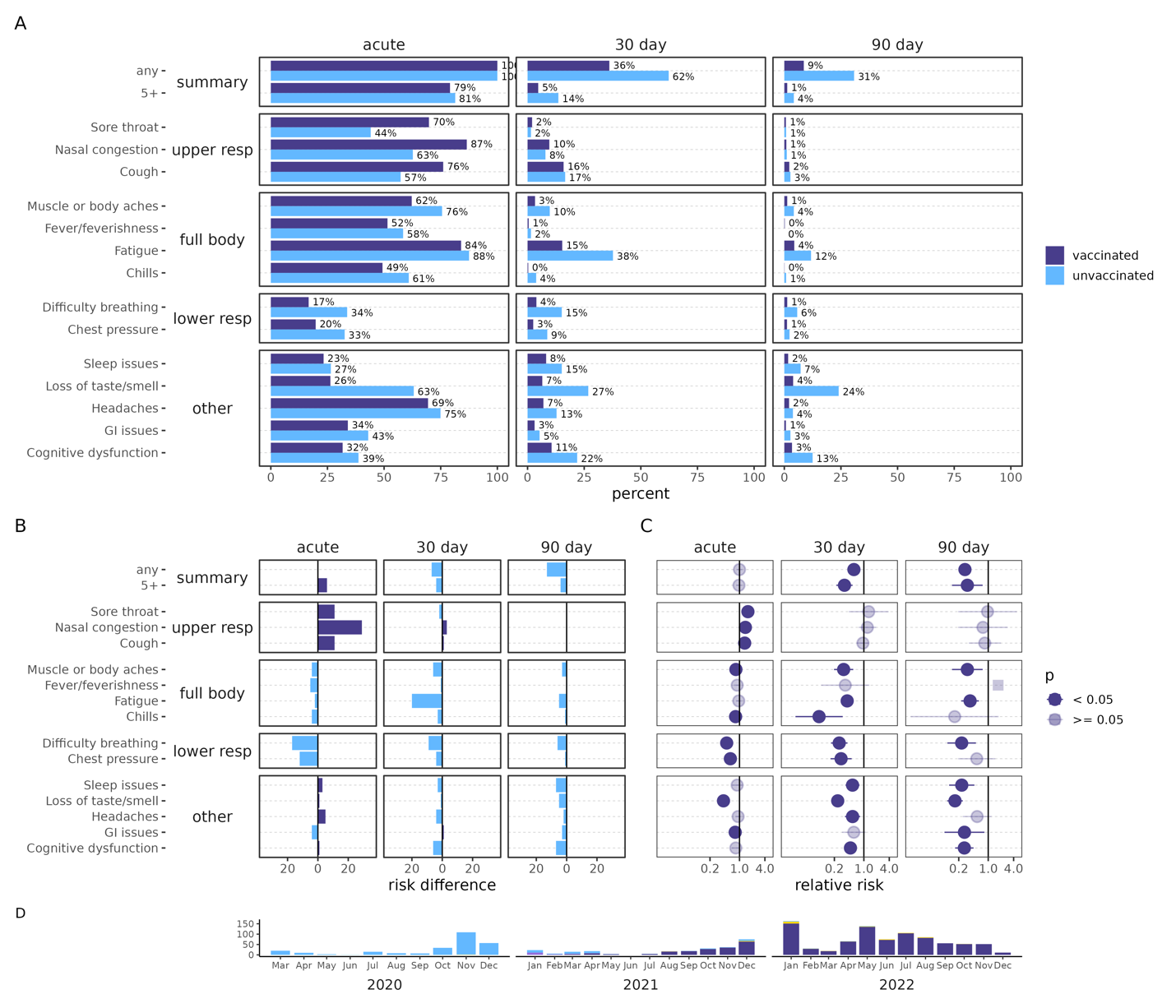


**Figure S13 Sensitivity analysis excluding acute asymptomatic cases for: Acute and PASC symptom prevalence, by prior immunity, among all cases.** Unvaccinated are in light blue and vaccinated in dark blue. A) Percentages reporting symptoms. B) Percentage point difference in reported symptoms, colored by the group reporting more symptoms. C) Relative risks for vaccinated vs unvaccinated cases. Squares indicate relative risks with a 0 count of symptoms that were calculated by adding 0.5 to all cells. Greyed out symptoms indicate comparisons that could not be made due to both groups reporting 0 symptoms. D) Plot of the subset of cases over time included in comparisons.

| Table S1. Symptom prevalence ratios for vaccinated compared to unvaccinated among all cases | | | | | | | | | | | | | | | | | | |
| --- | --- | --- | --- | --- | --- | --- | --- | --- | --- | --- | --- | --- | --- | --- | --- | --- | --- | --- |
|  |  | Acute (992 vaccinated, 321 unvaccinated) | | | | |  | PASC 30 days (978 vaccinated, 307 unvaccinated) | | | | |  | PASC 90 days (889 vaccinated, 305 unvaccinated) | | | | |
| Symptom Group | Symptom | RR | CI | f | S_e | S_u |  | RR | CI | f | S_e | S_u |  | RR | CI | f | S_e | S_u |
| summary | any | 1.09 | (0.95, 1.24) |  | 938 | 279 |  | 0.63 | (0.52, 0.76) |  | 330 | 165 |  | 0.31 | (0.22, 0.42) |  | 72 | 81 |
|  | 5+ | 1.06 | (0.91, 1.23) |  | 742 | 227 |  | 0.37 | (0.24, 0.58) |  | 43 | 36 |  | 0.34 | (0.15, 0.79) |  | 11 | 11 |
| upper resp | Sore throat | 1.72 | (1.42, 2.09) |  | 655 | 123 |  | 1.41 | (0.48, 4.16) |  | 18 | 4 |  | 1.03 | (0.21, 5.12) |  | 6 | 2 |
|  | Nasal congestion | 1.50 | (1.27, 1.77) |  | 811 | 175 |  | 1.31 | (0.81, 2.11) |  | 88 | 21 |  | 0.80 | (0.21, 3.11) |  | 7 | 3 |
|  | Cough | 1.44 | (1.22, 1.71) |  | 714 | 160 |  | 1.03 | (0.73, 1.44) |  | 145 | 44 |  | 0.89 | (0.37, 2.12) |  | 18 | 7 |
| full body | Fatigue | 1.05 | (0.91, 1.21) |  | 788 | 244 |  | 0.44 | (0.34, 0.56) |  | 140 | 100 |  | 0.40 | (0.25, 0.64) |  | 36 | 31 |
|  | Chills | 0.88 | (0.74, 1.05) |  | 462 | 170 |  | 0.09 | (0.03, 0.34) |  | 3 | 10 |  | 0.17 | (0.02, 1.89) |  | 1 | 2 |
|  | Muscle or body aches | 0.89 | (0.76, 1.05) |  | 583 | 211 |  | 0.36 | (0.21, 0.61) |  | 30 | 26 |  | 0.34 | (0.15, 0.79) |  | 11 | 11 |
|  | Fever/feverishness | 0.96 | (0.80, 1.15) |  | 483 | 163 |  | 0.39 | (0.11, 1.46) |  | 5 | 4 |  | 1.72 |  | 1.00 | 2 | 0 |
| lower resp | Difficulty breathing | 0.54 | (0.42, 0.70) |  | 157 | 94 |  | 0.28 | (0.18, 0.44) |  | 36 | 40 |  | 0.25 | (0.12, 0.55) |  | 11 | 15 |
|  | Chest pressure | 0.66 | (0.51, 0.85) |  | 186 | 91 |  | 0.31 | (0.18, 0.56) |  | 23 | 23 |  | 0.57 | (0.21, 1.58) |  | 10 | 6 |
| other | Sleep issues | 0.96 | (0.74, 1.25) |  | 219 | 74 |  | 0.58 | (0.40, 0.86) |  | 75 | 40 |  | 0.25 | (0.13, 0.50) |  | 14 | 19 |
|  | Loss of taste/smell | 0.45 | (0.37, 0.55) |  | 247 | 176 |  | 0.26 | (0.18, 0.37) |  | 59 | 71 |  | 0.18 | (0.11, 0.27) |  | 32 | 63 |
|  | Headaches | 1.01 | (0.86, 1.18) |  | 651 | 209 |  | 0.59 | (0.39, 0.89) |  | 64 | 34 |  | 0.59 | (0.27, 1.28) |  | 17 | 10 |
|  | GI issues | 0.86 | (0.70, 1.06) |  | 319 | 120 |  | 0.63 | (0.33, 1.19) |  | 28 | 14 |  | 0.29 | (0.10, 0.87) |  | 6 | 7 |
|  | Cognitive dysfunction | 0.89 | (0.71, 1.11) |  | 297 | 108 |  | 0.52 | (0.38, 0.72) |  | 97 | 58 |  | 0.30 | (0.18, 0.50) |  | 29 | 33 |
| RR = risk ratio (when there were 0 counts of symptoms, RR was calculated by adding 0.5 to all cells, otherwise using Poisson regression models) | | | | | | | | | | | | | | | |  |  |  |
| CI = confidence interval | |  |  |  |  |  |  |  |  |  |  |  |  |  |  |  |  |  |
| S_e = symptoms in the exposed group | | |  |  |  |  |  |  |  |  |  |  |  |  |  |  |  |  |
| S_u = symptoms in the unexposed group | | |  |  |  |  |  |  |  |  |  |  |  |  |  |  |  |  |
| f = p-value from Fisher's exact test (run when there were 0 counts of symptoms) | | | | | | | |  |  |  |  |  |  |  |  |  |  |  |

| Table S2. Symptom prevalence ratios for vaccinated compared to unvaccinated among pre-omicron cases | | | | | | | | | | | | | | | | | | |
| --- | --- | --- | --- | --- | --- | --- | --- | --- | --- | --- | --- | --- | --- | --- | --- | --- | --- | --- |
|  |  | Acute (144 vaccinated, 311 unvaccinated) | | | | |  | PASC 30 days (138 vaccinated, 297 unvaccinated) | | | | |  | PASC 90 days (129 vaccinated, 295 unvaccinated) | | | | |
| Symptom Group | Symptom | RR | CI | f | S_e | S_u |  | RR | CI | f | S_e | S_u |  | RR | CI | f | S_e | S_u |
| summary | any | 1.11 | (0.90, 1.36) |  | 138 | 269 |  | 0.99 | (0.75, 1.30) |  | 74 | 161 |  | 0.65 | (0.41, 1.03) |  | 23 | 81 |
|  | 5+ | 1.18 | (0.95, 1.48) |  | 120 | 219 |  | 0.78 | (0.41, 1.47) |  | 13 | 36 |  | 0.21 | (0.03, 1.61) |  | 1 | 11 |
| upper resp | Sore throat | 1.38 | (1.04, 1.85) |  | 75 | 117 |  | 0.24 |  | 0.31 | 0 | 4 |  | 1.14 | (0.10, 12.61) |  | 1 | 2 |
|  | Nasal congestion | 1.61 | (1.28, 2.03) |  | 126 | 169 |  | 1.53 | (0.79, 2.97) |  | 15 | 21 |  | 0.76 | (0.08, 7.33) |  | 1 | 3 |
|  | Cough | 1.32 | (1.03, 1.71) |  | 95 | 155 |  | 1.20 | (0.73, 1.98) |  | 24 | 43 |  | 0.98 | (0.25, 3.79) |  | 3 | 7 |
| full body | Fatigue | 1.08 | (0.86, 1.35) |  | 117 | 234 |  | 0.53 | (0.34, 0.83) |  | 24 | 97 |  | 0.66 | (0.32, 1.39) |  | 9 | 31 |
|  | Chills | 1.03 | (0.79, 1.35) |  | 78 | 163 |  | 0.21 | (0.03, 1.68) |  | 1 | 10 |  | 0.46 |  | 1.00 | 0 | 2 |
|  | Muscle or body aches | 1.05 | (0.82, 1.33) |  | 98 | 202 |  | 0.43 | (0.16, 1.12) |  | 5 | 25 |  | 0.42 | (0.09, 1.88) |  | 2 | 11 |
|  | Fever/feverishness | 1.02 | (0.77, 1.34) |  | 74 | 157 |  | 0.31 |  | 0.55 | 0 | 3 |  | 1.00 |  |  | 0 | 0 |
| lower resp | Difficulty breathing | 0.58 | (0.37, 0.90) |  | 25 | 93 |  | 0.49 | (0.24, 1.00) |  | 9 | 40 |  | 0.07 |  | 0.01 | 0 | 15 |
|  | Chest pressure | 0.72 | (0.48, 1.09) |  | 30 | 90 |  | 0.66 | (0.28, 1.53) |  | 7 | 23 |  | 0.76 | (0.15, 3.78) |  | 2 | 6 |
| other | Sleep issues | 1.22 | (0.83, 1.79) |  | 40 | 71 |  | 0.91 | (0.52, 1.61) |  | 17 | 40 |  | 0.12 | (0.02, 0.90) |  | 1 | 19 |
|  | Loss of taste/smell | 1.12 | (0.87, 1.45) |  | 91 | 175 |  | 1.09 | (0.73, 1.63) |  | 36 | 71 |  | 0.93 | (0.59, 1.48) |  | 25 | 63 |
|  | Headaches | 1.18 | (0.93, 1.49) |  | 109 | 200 |  | 0.81 | (0.43, 1.54) |  | 13 | 34 |  | 0.69 | (0.19, 2.49) |  | 3 | 10 |
|  | GI issues | 1.01 | (0.73, 1.39) |  | 54 | 116 |  | 1.23 | (0.52, 2.93) |  | 8 | 14 |  | 0.15 |  | 0.11 | 0 | 7 |
|  | Cognitive dysfunction | 1.13 | (0.82, 1.57) |  | 55 | 105 |  | 0.81 | (0.50, 1.33) |  | 22 | 58 |  | 0.55 | (0.26, 1.20) |  | 8 | 33 |
| RR = risk ratio (when there were 0 counts of symptoms, RR was calculated by adding 0.5 to all cells, otherwise using Poisson regression models) | | | | | | | | | | | | | | | |  |  |  |
| CI = confidence interval | |  |  |  |  |  |  |  |  |  |  |  |  |  |  |  |  |  |
| S_e = symptoms in the exposed group | | |  |  |  |  |  |  |  |  |  |  |  |  |  |  |  |  |
| S_u = symptoms in the unexposed group | | |  |  |  |  |  |  |  |  |  |  |  |  |  |  |  |  |
| f = p-value from Fisher's exact test (run when there were 0 counts of symptoms) | | | | | | | |  |  |  |  |  |  |  |  |  |  |  |

| Table S3. Symptom prevalence ratios for Omicron compared to pre-Omicron variant among vaccinated cases | | | | | | | | | | | | | | | | | | |
| --- | --- | --- | --- | --- | --- | --- | --- | --- | --- | --- | --- | --- | --- | --- | --- | --- | --- | --- |
|  |  | Acute (848 omicron, 144 pre-omicron variant) | | | | |  | PASC 30 days (840 omicron, 138 pre-omicron variant) | | | | |  | PASC 90 days (760 omicron, 129 pre-omicron variant) | | | | |
| Symptom Group | Symptom | RR | CI | f | S_e | S_u |  | RR | CI | f | S_e | S_u |  | RR | CI | f | S_e | S_u |
| summary | any | 0.98 | (0.82, 1.18) |  | 800 | 138 |  | 0.57 | (0.44, 0.74) |  | 256 | 74 |  | 0.36 | (0.22, 0.59) |  | 49 | 23 |
|  | 5+ | 0.88 | (0.72, 1.07) |  | 622 | 120 |  | 0.38 | (0.20, 0.73) |  | 30 | 13 |  | 1.70 | (0.22, 13.26) |  | 10 | 1 |
| upper resp | Sore throat | 1.31 | (1.03, 1.67) |  | 580 | 75 |  | 6.10 |  | 0.09 | 18 | 0 |  | 0.85 | (0.10, 7.29) |  | 5 | 1 |
|  | Nasal congestion | 0.92 | (0.76, 1.12) |  | 685 | 126 |  | 0.80 | (0.46, 1.39) |  | 73 | 15 |  | 1.02 | (0.12, 8.49) |  | 6 | 1 |
|  | Cough | 1.11 | (0.89, 1.37) |  | 619 | 95 |  | 0.82 | (0.53, 1.27) |  | 121 | 24 |  | 0.85 | (0.25, 2.95) |  | 15 | 3 |
| full body | Fatigue | 0.97 | (0.80, 1.19) |  | 671 | 117 |  | 0.79 | (0.51, 1.22) |  | 116 | 24 |  | 0.51 | (0.24, 1.08) |  | 27 | 9 |
|  | Chills | 0.84 | (0.66, 1.07) |  | 384 | 78 |  | 0.33 | (0.03, 3.62) |  | 2 | 1 |  | 0.51 |  | 1.00 | 1 | 0 |
|  | Muscle or body aches | 0.84 | (0.68, 1.04) |  | 485 | 98 |  | 0.82 | (0.31, 2.13) |  | 25 | 5 |  | 0.76 | (0.17, 3.54) |  | 9 | 2 |
|  | Fever/feverishness | 0.94 | (0.73, 1.20) |  | 409 | 74 |  | 1.81 |  | 1.00 | 5 | 0 |  | 0.85 |  | 1.00 | 2 | 0 |
| lower resp | Difficulty breathing | 0.90 | (0.58, 1.37) |  | 132 | 25 |  | 0.49 | (0.23, 1.04) |  | 27 | 9 |  | 3.92 |  | 0.38 | 11 | 0 |
|  | Chest pressure | 0.88 | (0.60, 1.31) |  | 156 | 30 |  | 0.37 | (0.15, 0.91) |  | 16 | 7 |  | 0.68 | (0.14, 3.21) |  | 8 | 2 |
| other | Sleep issues | 0.76 | (0.54, 1.07) |  | 179 | 40 |  | 0.56 | (0.32, 0.96) |  | 58 | 17 |  | 2.21 | (0.29, 16.87) |  | 13 | 1 |
|  | Loss of taste/smell | 0.29 | (0.22, 0.38) |  | 156 | 91 |  | 0.10 | (0.06, 0.18) |  | 23 | 36 |  | 0.05 | (0.02, 0.11) |  | 7 | 25 |
|  | Headaches | 0.84 | (0.69, 1.04) |  | 542 | 109 |  | 0.64 | (0.35, 1.18) |  | 51 | 13 |  | 0.80 | (0.23, 2.77) |  | 14 | 3 |
|  | GI issues | 0.83 | (0.62, 1.12) |  | 265 | 54 |  | 0.41 | (0.18, 0.93) |  | 20 | 8 |  | 2.21 |  | 0.60 | 6 | 0 |
|  | Cognitive dysfunction | 0.75 | (0.56, 1.00) |  | 242 | 55 |  | 0.56 | (0.35, 0.90) |  | 75 | 22 |  | 0.45 | (0.20, 1.01) |  | 21 | 8 |
| RR = risk ratio (when there were 0 counts of symptoms, RR was calculated by adding 0.5 to all cells, otherwise using Poisson regression models) | | | | | | | | | | | | | | | |  |  |  |
| CI = confidence interval | |  |  |  |  |  |  |  |  |  |  |  |  |  |  |  |  |  |
| S_e = symptoms in the exposed group | | |  |  |  |  |  |  |  |  |  |  |  |  |  |  |  |  |
| S_u = symptoms in the unexposed group | | |  |  |  |  |  |  |  |  |  |  |  |  |  |  |  |  |
| f = p-value from Fisher's exact test (run when there were 0 counts of symptoms) | | | | | | | |  |  |  |  |  |  |  |  |  |  |  |

| Table S4. Symptom prevalence ratios for prior infected compared to prior uninfected among vaccinated cases | | | | | | | | | | | | | | | | | | |
| --- | --- | --- | --- | --- | --- | --- | --- | --- | --- | --- | --- | --- | --- | --- | --- | --- | --- | --- |
|  |  | Acute (98 prior infection, 894 first infection) | | | | |  | PASC 30 days (96 prior infection, 882 first infection) | | | | |  | PASC 90 days (85 prior infection, 804 first infection) | | | | |
| Symptom Group | Symptom | RR | CI | f | S_e | S_u |  | RR | CI | f | S_e | S_u |  | RR | CI | f | S_e | S_u |
| summary | any | 0.97 | (0.78, 1.20) |  | 90 | 848 |  | 1.06 | (0.74, 1.50) |  | 34 | 296 |  | 1.18 | (0.57, 2.47) |  | 8 | 64 |
|  | 5+ | 0.95 | (0.74, 1.22) |  | 70 | 672 |  | 2.78 | (1.37, 5.65) |  | 10 | 33 |  | 2.10 | (0.45, 9.73) |  | 2 | 9 |
| upper resp | Sore throat | 1.06 | (0.82, 1.36) |  | 68 | 587 |  | 0.54 | (0.07, 4.05) |  | 1 | 17 |  | 0.72 |  | 1.00 | 0 | 6 |
|  | Nasal congestion | 0.96 | (0.76, 1.21) |  | 77 | 734 |  | 1.31 | (0.70, 2.47) |  | 11 | 77 |  | 1.59 | (0.19, 13.22) |  | 1 | 6 |
|  | Cough | 0.99 | (0.77, 1.27) |  | 70 | 644 |  | 1.14 | (0.68, 1.91) |  | 16 | 129 |  | 1.19 | (0.27, 5.18) |  | 2 | 16 |
| full body | Fatigue | 0.95 | (0.74, 1.20) |  | 74 | 714 |  | 1.44 | (0.89, 2.34) |  | 19 | 121 |  | 2.28 | (1.00, 5.21) |  | 7 | 29 |
|  | Chills | 0.75 | (0.53, 1.06) |  | 35 | 427 |  | 1.31 |  | 1.00 | 0 | 3 |  | 28.23 |  | 0.10 | 1 | 0 |
|  | Muscle or body aches | 0.95 | (0.72, 1.25) |  | 55 | 528 |  | 1.02 | (0.31, 3.36) |  | 3 | 27 |  | 3.54 | (0.94, 13.35) |  | 3 | 8 |
|  | Fever/feverishness | 0.65 | (0.45, 0.93) |  | 32 | 451 |  | 2.30 | (0.26, 20.55) |  | 1 | 4 |  | 9.45 | (0.59, 151.01) | | 1 | 1 |
| lower resp | Difficulty breathing | 1.41 | (0.89, 2.23) |  | 21 | 136 |  | 1.84 | (0.76, 4.41) |  | 6 | 30 |  | 2.10 | (0.45, 9.73) |  | 2 | 9 |
|  | Chest pressure | 1.62 | (1.08, 2.42) |  | 28 | 158 |  | 1.93 | (0.66, 5.68) |  | 4 | 19 |  | 4.10 | (1.06, 15.84) |  | 3 | 7 |
| other | Sleep issues | 1.12 | (0.73, 1.72) |  | 24 | 195 |  | 1.58 | (0.83, 2.99) |  | 11 | 64 |  | 5.25 | (1.76, 15.68) |  | 5 | 9 |
|  | Loss of taste/smell | 0.89 | (0.58, 1.38) |  | 22 | 225 |  | 0.85 | (0.34, 2.12) |  | 5 | 54 |  | 0.63 | (0.15, 2.65) |  | 2 | 30 |
|  | Headaches | 0.99 | (0.77, 1.29) |  | 64 | 587 |  | 1.91 | (1.00, 3.65) |  | 11 | 53 |  | 3.98 | (1.40, 11.29) |  | 5 | 12 |
|  | GI issues | 1.16 | (0.82, 1.64) |  | 36 | 283 |  | 1.99 | (0.76, 5.24) |  | 5 | 23 |  | 1.89 | (0.22, 16.19) |  | 1 | 5 |
|  | Cognitive dysfunction | 1.26 | (0.89, 1.78) |  | 36 | 261 |  | 1.68 | (0.97, 2.91) |  | 15 | 82 |  | 1.97 | (0.75, 5.16) |  | 5 | 24 |
| RR = risk ratio (when there were 0 counts of symptoms, RR was calculated by adding 0.5 to all cells, otherwise using Poisson regression models) | | | | | | | | | | | | | | | |  |  |  |
| CI = confidence interval | |  |  |  |  |  |  |  |  |  |  |  |  |  |  |  |  |  |
| S_e = symptoms in the exposed group | | |  |  |  |  |  |  |  |  |  |  |  |  |  |  |  |  |
| S_u = symptoms in the unexposed group | | |  |  |  |  |  |  |  |  |  |  |  |  |  |  |  |  |
| f = p-value from Fisher's exact test (run when there were 0 counts of symptoms) | | | | | | | |  |  |  |  |  |  |  |  |  |  |  |
